# Electroconductive MXene‐Thin‐Film‐Composite Membranes for Coupled Osmotic Concentration and PFAS Mineralization

**DOI:** 10.1002/smll.75540

**Published:** 2026-08-31

**Authors:** Afrouz Yousefi, Pooria Karami, Vahid Rad, Amir Aghaei, Mojtaba Mohseni, Lingling Yang, Masoud Soroush, Matthias Wessling, Mohamed Gamal El‐Din, Mohtada Sadrzadeh

**Affiliations:** ^1^ Department of Mechanical Engineering 10–241 Donadeo Innovation Center for Engineering Advanced Water Research Lab (AWRL) University of Alberta Edmonton Alberta Canada; ^2^ Department of Civil and Environmental Engineering University of Alberta Edmonton Alberta Canada; ^3^ Department of Chemical and Biological Engineering Drexel University Philadelphia Pennsylvania USA; ^4^ RWTH Aachen University Aachener Verfahrenstechnik – Chemical Process Engineering Aachen Germany; ^5^ Department of Materials Science and Engineering Drexel University Philadelphia Pennsylvania USA

**Keywords:** advanced oxidation, forward osmosis, MXene, perfluorooctanoic acid degredation, per‐ and polyfluoroalkyl substances, thin‐film composite membranes

## Abstract

We introduce an integrated treatment system that couples osmotic dewatering with advanced oxidation (AO) using electrically conductive Ti_3_C_2_T*_x_* MXene‐based membranes, enabling simultaneous PFAS concentration and electrochemical degradation and mineralization. Conductive thin‐film composite membranes were fabricated by depositing Ti_3_C_2_T*_x_* either on the active layer or on the porous support, with the active layer retained on the opposite surface. These architectures enabled operation of advanced‐ oxidation forward‐osmosis (AO‐FO) and advanced‐ oxidation pressure‐retarded‐osmosis (AO‐PRO) modes, respectively. The MXene‐based membranes exhibited high electrical conductivity (5446 S·cm^−1^) and achieved 99% rejection of perfluorooctanoic acid (PFOA). Electrochemical degradation experiments showed that increasing the applied current density from 200 to 500 A/m^2^ enhanced PFOA degradation and mineralization. At 500 A/m^−2^, total organic carbon removal reached 75% in the AO‐FO mode and 93% in the AO‐PRO mode. The superior performance of the AO‐PRO configuration is attributed to the synergistic effects of stronger osmotic dewatering, which increases the local PFOA concentration, and the favorable structural and electrochemical properties of the MX‐B membrane architecture, including lower charge‐transfer resistance and enhanced physical stability, thereby accelerating PFOA degradation. Furthermore, the integrated process reduced energy consumption, highlighting the potential of MXene‐enabled osmotic‐electrochemical systems for energy‐efficient PFAS destruction and safe effluent management.

## Graphical abstract

An electroconductive MXene‐TFC membrane integrates osmotic dewatering with anodic oxidation to concentrate and mineralize PFOA at the membrane interface. The coupled AO‐PRO configuration accelerates degradation kinetics, achieves 99% rejection and up to 93% TOC removal, and lowers specific energy demand, offering a compact route for PFAS destruction.

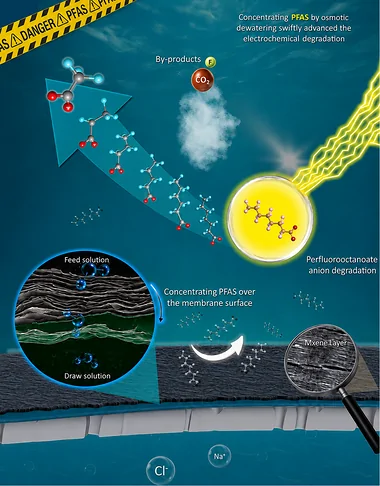

## 1 Introduction

Per‐ and polyfluoroalkyl substances (PFAS) constitute a large family of synthetic organo‐fluorine compounds, widely used in industrial and consumer products such as firefighting foams, food packaging materials, and fluoropolymer manufacturing, owing to their exceptional thermal and chemical stability [1, 2]. The strength of the carbon‐fluorine (C─F) bonds renders PFAS highly resistant to biodegradation, hydrolysis, and photolysis; together with their water solubility and low volatility, these properties result in environmental persistence, mobility, and bioaccumulation in biota [3, 4]. Among PFAS, perfluorooctanoic acid (PFOA) is a frequently detected long‐chain species in wastewater, drinking water, and seawater [5, 6]. Owing to documented health risks and the persistence of these compounds, regulatory limits have become increasingly strict. The U.S. Environmental Protection Agency (EPA) recently finalized its National Primary Drinking Water Regulations (NPDWR), replacing previous advisories with a legally enforceable Maximum Contaminant Level (MCL) of just 4.0 ng L^−^^1^ for PFOA. Similarly, the 2024 update to the Guidelines for Canadian Drinking Water Quality established a highly stringent new objective, capping the sum of detected PFAS at 30 ng L^−1^ [7]. Therefore, developing effective PFAS removal in water systems has become a global concern for protecting both environmental and human health.

Researchers have investigated a wide range of destructive and non‐destructive technologies for treating PFAS‐contaminated waters, including adsorption [8, 9, 10], membrane separation [11, 12, 13, 14, 15, 16, 17], electrochemical processes [18, 19, 20], microwave‐assisted treatments [21, 22], and photocatalysis [23, 24]. Adsorption is straightforward and scalable, but regenerating spent sorbents produces a PFAS‐rich solution that requires further treatment [8, 25, 26]. Pressure‐driven membranes, such as nanofiltration and reverse osmosis, effectively reject PFAS yet require high electrical energy and produce concentrated PFAS brines that must be managed [16]. Photocatalysis can achieve high removal but depends on specialized catalysts and controlled irradiation, complicating scale‐up [27, 28]. Among advanced oxidation processes (AOPs), electrochemical oxidation, including anodic oxidation (AO) and cathode‐mediated routes (e.g., electro‐Fenton via H_2_O_2_ at the cathode, persulfate activation, photo‐electro‐oxidation), has emerged as a promising destructive option for PFAS by generating reactive species that attack PFAS molecules [29, 30]. However, even with reported removal rates>90%, electrochemical oxidation is energy‐intensive because hydroxyl radicals (•OH) are relatively ineffective at complete PFOA mineralization, especially at low concentrations (µg L^−1^‐ng L^−1^) [31]. At low initial PFOA concentrations (<100 mg L^−^^1^), rapid depletion near the anode, coupled with slow bulk replenishment, resulted in diffusion‐limited kinetics and reduced pseudo‐first‐order rate constants [32]. Similarly, electrochemical oxidation of PFOA and perfluorooctane sulfonate (PFOS) at trace concentrations showed low defluorination efficiency and long reaction times; even higher current density could not overcome the mass‐transfer bottleneck, so mineralization rates declined significantly as concentrations decreased [33, 34].

These challenges highlight the need for treatment approaches that lower energy use while still achieving effective PFAS destruction. A practical pathway is a hybrid concentrate‐and‐destroy approach: first employ a high‐rejection separation to enrich PFAS, then apply a destructive step at higher pollutant activity where advanced oxidation is more efficient and less energy‐intensive. In our prior work, we integrated membrane distillation (MD) with electro‐Fenton (EF) and AO to remove PFOA. Continuous dewatering by MD accelerated EF/AO kinetics and improved overall removal, with electrodes placed in the feed chamber being simultaneously concentrated by the MD cell [35]. A similar dewatering step can also be achieved using forward osmosis (FO), which uses an osmotic pressure gradient to draw water from a low‐osmotic feed to a high‐osmotic draw solution across a dense semipermeable membrane [36, 37, 38]. Operating without applied hydraulic pressure, FO typically reduces energy demand and mitigates fouling relative to pressure‐driven processes [39]. By concentrating on PFAS, FO creates favorable conditions for subsequent chemical degradation. Beyond sequential trains, an alternative strategy is to embed electrochemical functionality within the membrane, enabling in situ concentration and destruction, thereby further reducing energy and footprint requirements.

Recent developments in electroconductive membranes (ECMs) enable co‐located separation and electrochemical oxidation within a single architecture, offering a compact, energy‐efficient route for PFAS treatment. ECMs can be fabricated either by bulk incorporation of conductive fillers or polymers or by post‐fabrication surface functionalization of porous supports. A main challenge for surface‐modified ECMs is achieving sufficient electrical conductivity without compromising mass transfer: forming a continuous, highly conductive coating often requires thicker or denser coatings that increase hydraulic resistance. Equally critical is long‐term electrochemical, mechanical, and physicochemical stability to maintain performance over extended operation [14]. A variety of materials have been explored for ECM fabrication, including carbon nanotubes, graphene derivatives, conductive polymers, metal oxides, and emerging 2D materials such as MXene, each offering distinct advantages in conductivity, stability, and processability. While carbon‐based nanomaterials such as graphene and carbon nanotubes (CNTs) exhibit high theoretical conductivity, their intrinsic hydrophobicity and tendency to aggregate in aqueous environments often necessitate complex surface functionalization. This functionalization frequently disrupts their sp^2^‐hybridized carbon networks, creating a severe trade‐off between aqueous processability and electrical performance. Conversely, conductive polymers offer favorable dispersibility but are highly susceptible to rapid degradation when exposed to potent reactive oxygen species (e.g., •OH) generated during anodic oxidation. In contrast, Ti_3_C_2_T_x_ MXene uniquely overcomes these limitations. Its abundant surface terminations (─OH, = O, ─F) render it intrinsically hydrophilic, ensuring excellent aqueous dispersibility, membrane wettability, and optimal ion transport without compromising its structural integrity. Furthermore, MXene retains an exceptional metallic‐level electrical conductivity alongside robust electrochemical stability, making it a superior choice for integrating continuous osmotic separation with aggressive advanced oxidation environments [40].

MXenes are a class of 2D transition‐metal carbides and/or nitrides with the general formula M_n+1_X_n_T*_x_* (*n* = 1–4), where M is an early transition metal, X is carbon and/or nitrogen, and 𝑇_x_ denotes surface terminations (e.g., ═O, ─OH, ─F, ─Cl) [40]. Their lamellar structure, tunable interlayer spacing, intrinsic hydrophilicity, and adaptable surface chemistry make them compelling for membrane‐based separations. Among MXenes, Ti_3_C_2_T*_x_* is the most extensively studied, with a substantial body of work detailing its synthesis, structural characteristics, and transport behavior [41]. Its surface terminations facilitate covalent bonding, hydrogen bonding, dipole‐dipole interactions, and electrostatic interactions with host polymers, improving interfacial compatibility and enabling uniform dispersion within composite membranes [42]. In multilayer form, Ti_3_C_2_T*_x_* MXene also exhibits outstanding electrical conductivity, up to 24 000 S cm^−1^, supporting applications across separations and energy systems [43].

While recent studies have demonstrated electroconductive MXene‐coated membranes for coupled PFAS electrosorption and mineralization via hydraulic filtration [44, 45], integrating these conductive architectures into osmotically driven membrane systems (FO and PRO) remains unexplored. Unlike hydraulic filtration, osmotically driven configurations offer a unique synergy by continuously concentrating PFAS at the membrane boundary without external hydraulic pressure, enhancing local reaction kinetics at the electroactive interface. This study introduces a novel PFAS treatment approach by integrating osmotic dewatering and AO into a single, simultaneous process. Rather than relying on energy‐intensive sequential treatment trains, we developed a dual‐function Ti_3_C_2_T_x_ MXene‐based thin‐film composite (TFC) membrane that operates concurrently as a selective barrier and an electrocatalytic anode. Driven by a natural osmotic pressure gradient, the system actively draws water across the membrane, concentrating dilute PFOA molecules directly at the active anode interface. This concentration effect dramatically accelerates AO kinetics. This hybrid platform achieves rapid PFOA mineralization with significantly reduced overall energy consumption. In this work, we extensively characterized the fabricated conductive MXene membranes, evaluated their efficacy for PFOA removal, and quantified the influence of applied current density on degradation kinetics under standalone AO versus hybrid FO‐AO/PRO operation.

## 2 Experimental Section

### 2.1 Material

Titanium powder (−325 mesh, 99.5%), titanium carbide (99.5%), aluminum powder (−325 mesh, 99.5%), Lithium chloride (LiCl), hydrochloric acid (HCl, 37%, ACS grade), hydrofluoric acid (HF, 48%, ACS grade), and carboxymethyl cellulose sodium salt (CMC) were obtained from Thermo Fisher Scientific. Poly (vinyl alcohol) (PVA, 13 000–23 000 g/mol, 87%–89% hydrolyzed) was supplied by Sigma–Aldrich. Commercial polyethersulfone (PES, 0.2 µm pore size) membranes (Sterlitech, WA, USA) were used as support for FO membranes. Polyamide (PA) selective layers were formed via interfacial polymerization of m‐phenylenediamine (MPD, ≥99%, Sigma–Aldrich) and trimesoyl chloride (TMC, 98%, Sigma–Aldrich), using heptane (≥99%, Fisher Scientific) as the organic solvent. Sodium dodecyl sulfate (SDS) and triethylamine (TEA) (Sigma–Aldrich) were used as additives. All reagents were used without further purification.

Sodium sulfate (Na_2_SO_4_, Sigma–Aldrich) was used as the electrolyte because of its chemical stability, non‐toxicity, and ability to sustain hydroxyl radical generation as the dominant oxidant during anodic oxidation. Compared with sodium chloride (NaCl), which produces harmful chlorinated by‐products and corrodes electrodes, Na_2_SO_4_ is non‐corrosive, non‐scaling, and improves electrode durability and conductivity, reducing energy demand and enhancing PFAS degradation efficiency. Model PFAS solutions were prepared by dissolving PFOA (MW: 414.07 g/mol, Sigma–Aldrich), perfluorooctanesulfonic acid (PFOS, MW: 500.13 g/mol, Sigma–Aldrich), and perfluoropentanoic acid (PFPeA, MW: 264.04 g/mol, Sigma–Aldrich) in Milli‐Q water (18.2 MΩ·cm).

### 2.2 Synthesis of Ti_3_AlC_2_ MAX

The Ti_3_AlC_2_ MAX phase was synthesized from titanium, titanium carbide, and aluminum powders mixed in a molar ratio of 2:1.25:2.2 [46]. The precursor blend was subjected to ball milling for 18 h at 64 rpm in Nalgene high‐density polyethylene bottles using yttria‐stabilized zirconia balls, with a ball‐to‐powder mass ratio of 2:1. The resulting powder was placed in an alumina crucible, wrapped with graphite foil, and heated in a Carbolite Gero tube furnace (1700°C model). Before heating, the furnace chamber was purged with high‐purity argon (99.999%) for 20 min. The sample was then heated to 1400°C at 3.5°C min^−1^, held for 4 h under continuous argon flow, and cooled to room temperature at 10°C min^−1^. The sintered block was finally ground to a fine powder (<71 µm) using a TiN‐coated milling bit.

### 2.3 Synthesis of Ti_3_C_2_T*_x_* MXene

Ti_3_C_2_T*_x_* MXene was synthesized according to our previously reported procedure [46]. Briefly, 1 g of the Ti_3_AlC_2_ MAX phase was added to 10 mL of 9 M HCl under stirring (400 rpm) for 18 h at room temperature to remove intermetallic impurities. The purified powder was then slowly added to 30 mL of an etchant solution (volume ratio: 6 parts 12 M HCl, 3 parts deionized water, 1 part 28.4 M HF) and stirred at 35°C and 400 rpm for 24 h. The etched suspension was repeatedly washed with deionized water (∼200 mL per cycle) by centrifugation (3234 g) until the supernatant reached ∼pH 6.

For delamination, the resulting multilayer Ti_3_C_2_T*_x_* sediment (50 mL per gram of MAX phase) was treated with 0.5 M LiCl solution and stirred at 65°C and 400 rpm for 1 h under an Ar atmosphere. The dispersion was centrifuged at 3234 g in sequential steps of 5, 10, 15, and 20 min to remove unexfoliated material. The collected supernatant was vortexed for 30 min and then centrifuged again (2380 g, 30 min) to obtain single‐ to few‐layer Ti_3_C_2_T*_x_* flakes. The final suspension was stored at −4°C until use [47].

### 2.4 Membrane Fabrication

#### 2.4.1 Preparation of Thin‐Film Composite Membranes

TFC membrane consists of a porous support layer coated with a dense, fully aromatic polyamide selective layer. These membranes were fabricated by interfacial polymerization of MPD and TMC at the interface between an aqueous and an organic solution. The aqueous phase contained 1.0 wt.% MPD, 0.2 wt.% SDS, and 1 wt.% TEA in water, and the organic phase contained 0.4 wt.% TMC in n‐heptane. PES substrates were first soaked in the aqueous solution for 5 min, excess solution was removed with an acrylic roller, and the soaked PES substrates were then fixed in a custom‐made holder (two plexiglass frames, 16 × 12 cm^2^, sealed with silicone rubber). The TMC solution was then poured onto the support, and the interfacial polymerization reaction was allowed to proceed for 30 s. The resulting membranes were cured in an oven at 60°C for 4 min, rinsed with DI water to remove unreacted monomers, and finally stored in DI water at room temperature.

#### 2.4.2 Preparation of MXene‐Modified TFC Membranes

Conductive TFC membranes were fabricated using two different approaches. In the first method, MXene suspensions containing Ti_3_C_2_T*_x_* and CMC were prepared in water and deposited onto the synthesized TFC membrane by vacuum filtration. CMC is used as a binder to ensure strong, stable adhesion between the MXene layers and the membrane. The coated membranes were subsequently cured in an oven at 60°C for 1 h to stabilize the coating layer.

In the second method, the MXene coating and the PA selective layer were deposited on two opposite sides of the PES support membrane. To improve interfacial adhesion between the PES support and the MXene layer, the substrate was first modified with a thin PVA coating. A 1 wt.% PVA solution was prepared by heating at 80°C for 2 h to achieve complete dissolution. The PES substrate was then dip‐coated with the PVA solution for 2 min. It was subsequently withdrawn at a controlled rate, leaving a thin liquid film on the surface. The coated substrate was dried at 60°C for 1 h to evaporate the solvent and form the film. A suspension of MXene and CMC was then deposited onto the PVA‐PES supports by vacuum filtration. The Ti_3_C_2_T_x_‐coated PES membranes were cured at 60°C for 15 min. Next, the PA selective layer was synthesized on the opposite side of the PES substrate via interfacial polymerization, as described in Section 2.4.1.

Figure 1 shows the fabrication steps of the TFC and MXene‐TFC composite membranes via interfacial polymerization and vacuum‐assisted coating. The two architectures were not introduced merely as alternative coating methods but were designed to enable two distinct coupled operating modes. In the MX‐T configuration, the MXene layer is located on the PA‐facing side to support the AO‐FO mode. In the MX‐B configuration, the MXene layer is deposited on the support side, while the PA layer is formed on the opposite side, enabling AO‐PRO operation and positioning the conductive MXene interface to enhance contact with the concentrated PFAS‐containing feed during osmotic dewatering. This architectural difference is central to the study because it allows us to compare how membrane orientation and osmotic driving force influence PFAS concentration, degradation kinetics, and mineralization.

**FIGURE 1:**
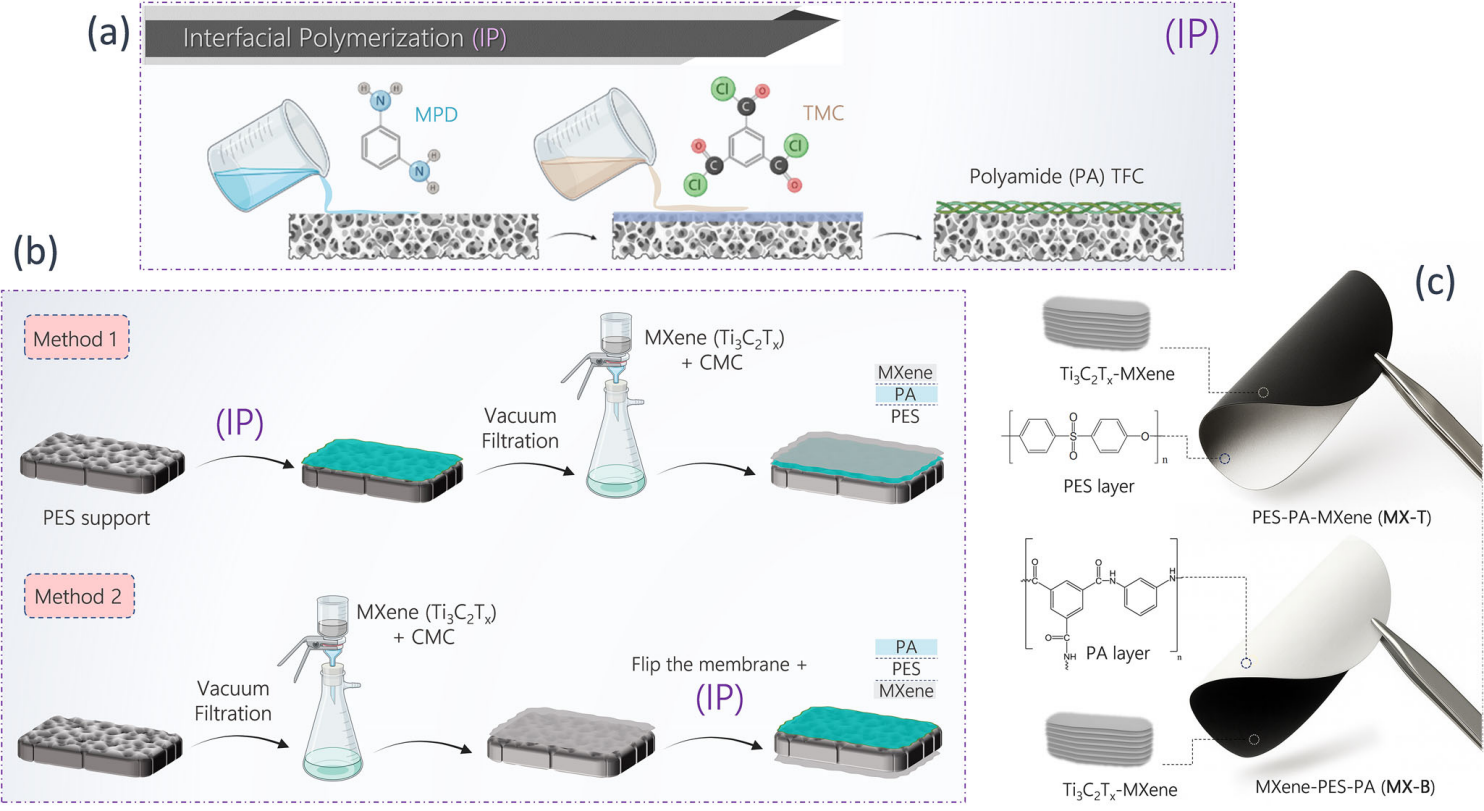
(a) Schematic illustration of TFC membrane fabrication through the interfacial polymerization reaction between MPD and TMC over the PES substrate, (b) Sequential steps for the preparation of MXene‐TFC membranes using two different methods: in *Method 1*, the PA layer was first formed by interfacial polymerization, followed by vacuum‐assisted deposition of the MXene/CMC suspension; in *Method 2*, the MXene/CMC layer was first deposited on the PES substrate, the membrane was then flipped, and the PA layer was subsequently formed on the opposite side, and (c) the structure of the resulting asymmetric composite membranes, showing MX‐T and MX‐B configurations.

### 2.5 Physicochemical Characterizations of MXene and Fabricated Membranes

Detailed physicochemical characterization procedures are provided in the Supporting Information. In brief, surface wettability of the pristine TFC and MXene‐TFC membranes was assessed by contact angle analysis. MXene powder and membrane morphologies were examined using field‐emission scanning electron microscopy (FESEM), while elemental composition was determined by energy‐dispersive spectroscopy (EDS). Transmission electron microscopy (TEM) was used to assess coating thickness and structural integrity. Zeta potential was measured by an electrokinetic analyzer, and surface roughness and topography were characterized by atomic force microscopy (AFM). Crystallinity was analyzed by x‐ray diffraction (XRD). Sheet resistivity was measured with a four‐point probe, and the membrane's electrical conductivity (S/cm) was calculated as 1/resistivity. Electrochemical behavior was investigated by linear sweep voltammetry (LSV) and cyclic voltammetry (CV) under N_2−_ and air‐saturated conditions. Finally, the stability of the MXene coating was evaluated by monitoring titanium ion release from the membranes.

### 2.6 Separation Performance of Synthesized Membranes in FO Operation

The separation performance of synthesized membranes was evaluated using a laboratory‐scale cross‐flow filtration setup. The membrane cell was designed with channels on both sides of the membrane. The length, width, and height of both channels were 39, 39, and 4 mm, respectively, providing an effective filtration area of 15 cm^2^. Both the feed and draw solution flow rates were maintained at 1 L·min^−1^. The performance of the synthesized TFC and MXene‐coated membranes was evaluated under two operational configurations: FO mode, with the polyamide active layer facing the feed solution (AL‐FS), and pressure‐retarded osmosis (PRO) mode, with the active layer oriented toward the draw solution (AL‐DS). The AL‐DS configuration was employed to determine the water permeability coefficient (A) and the salt permeability coefficient (B). Sodium chloride solutions of varying concentrations (0.5, 2.0 M, with 0.5 M increments) served as the draw solution, whereas deionized (DI) water was used as the feed to minimize the effect of concentrative internal concentration polarization (ICP). In this condition, ICP arises exclusively from the reverse salt flux into the feed, as no solute accumulation occurs from water transport [48]. The reverse salt flux (*J_s_*) was calculated from conductivity measurements of the feed at different time intervals, while the water flux (*J_w_*) was determined by monitoring changes in the draw solution mass [49].

(1) $$J_{w}=\frac{\Delta m}{\rho \;A\;\Delta t}$$

where Δ*m* is the change in the draw solution mass, Δ*t* is the time interval, *A* is the effective membrane surface area, and ρ is the density of water. The reverse salt flux (*J_s_*) was calculated using:

(2) $$J_{s}=\frac{\Delta \left(C_{t}V_{t}\right).\;M_{w}}{A.\Delta t}$$

where *C_t_* is the salt concentration at time *t*, *V_t_* is the feed solution volume at time *t*, and *M_w_* is the molar mass of salt.

### 2.7 Evaluating the FO and AO Hybrid Process

Figure 2 shows a schematic of the FO setup designed for electroconductive membranes. The setup integrates two simultaneous treatment processes: (i) osmotic dewatering and (ii) anodic oxidation (AO). Driven by the osmotic pressure gradient, water molecules naturally move from the low‐salinity PFAS‐containing feed solution toward the high‐salinity draw solution. This water flux results in PFAS concentration on the feed side, thereby enhancing the AO process efficiency by increasing the local pollutant concentration near the membrane surface. The electroconductive nature of MXene‐TFC membranes facilitates in situ generation of reactive oxidative species under an applied potential, accelerating PFAS degradation into smaller, less toxic molecules. Overall, this hybrid system enables simultaneous water extraction and contaminant breakdown in a single, energy‐efficient configuration. The PFAS degradation and removal were tested under three operational modes:

1. AO: The draw solution was sealed to block water flow, enabling the electroconductive membrane surface to function as the anode.
2. AO‐FO: The MX‐T TFC membrane was used with its active layer facing the feed solution, enabling simultaneous osmotic water transport and electrochemical oxidation.
3. AO‐PRO: The MX‐B TFC membrane was oriented with the PA layer facing the draw solution and the MXene layer facing the feed side, maximizing osmotic driving force and enhancing electrochemical degradation.

**FIGURE 2:**
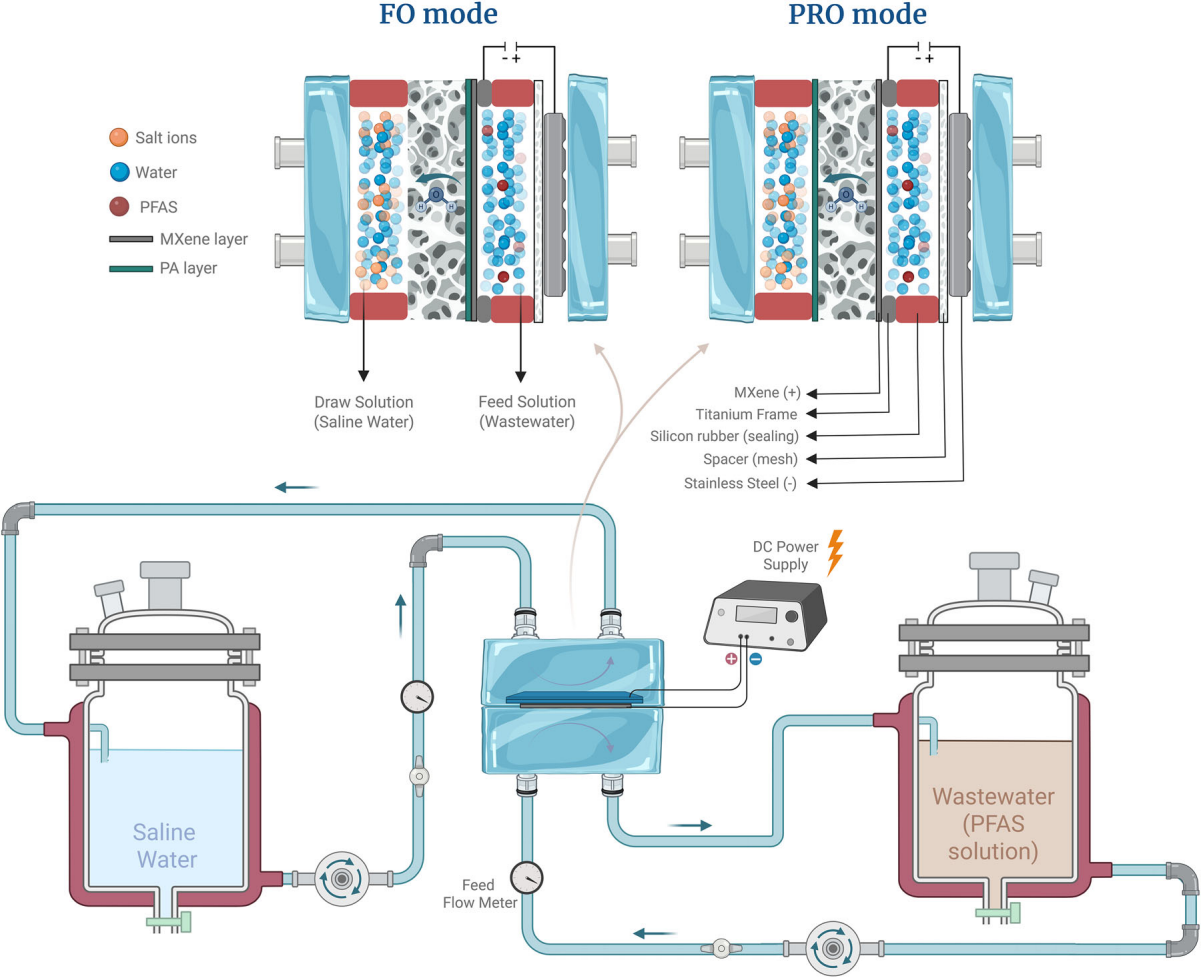
Schematic of FO setup designed for electroconductive membranes capable of operating in both FO and PRO modes for simultaneous osmotic dewatering and PFOA degradation. The electroconductive MXene‐based membrane, assembled between a stainless‐steel electrode and a titanium frame with silicone rubber sealing and a spacer mesh, enables in situ electrochemical degradation of PFOA under an applied DC voltage while maintaining high water permeability and selective ion transport.

For AO experiments, the MX‐T and MX‐B membranes were connected to the positive pole of a DC power supply (Wuxi Qiaowei Electronics Co., Ltd, China), while a stainless‐steel mesh (McMaster‐Carr, USA) served as the cathode. A silicone rubber frame and spacer were placed between the membrane and the mesh to prevent direct contact. The feed solution contained 20 ppm of PFOA as a model contaminant and 50 mM Na_2_SO_4_ as a supporting electrolyte. A 2 M NaCl solution was used as the draw solution. Both the feed and draw solutions were circulated at 1 L min^−1^. The osmotic dewatering increased the PFOA concentration in the feed solution. To correct for this concentration rise, the following equation was applied:

(3) $$C_{Corrected}=C_{Measured}\;\;\times \left(1-\frac{V_{Permeated}}{V_{Initial}}\right)$$

All experiments were performed in triplicate to ensure reliable, reproducible data. Following each run, all components of the setup, including the beaker, electrodes, and experimental platform, were thoroughly cleaned to maintain measurement accuracy and eliminate any risk of cross‐contamination.

The experimental setup is illustrated in Figure 2, which depicts a FO system equipped with a DC power supply for electroconductive MXene‐based membranes. The setup can operate in either FO or PRO mode depending on the orientation of the TFC membrane. The Figure also shows the membrane cell assembly, including stainless steel and titanium electrodes, silicone rubber sealing, and a spacer mesh that ensures proper electrical contact and water flow during operation.

### 2.8 Analytical methods

Details of the analytical methods, formulas, and calculations are provided in the Supporting Information.

## 3 Results and Discussion

### 3.1 Characterization of Ti_3_C_2_T*_x_* MXene Nanosheets

Figure 3a presents the surface morphology of the synthesized MXene nanosheets. SEM imaging reveals a layered structure with relatively smooth surfaces. Figure 3b depicts the elemental composition of MXene nanosheets determined by EDX analysis, indicating the presence of carbon (13.9%), oxygen (9.6%), titanium (54.2%), and fluorine (13.6%). Figure 3c presents HRTEM images of the synthesized MXene, showing its well‐defined crystalline structure. Additional TEM images at different magnifications are provided in Figure S3. XRD spectrum (Figure 3d) confirms the successful transition of Ti_3_AlC_2_ to Ti_3_C_2_T*_X_* MXene. The absence of MAX‐phase peaks (around 39)and the shift of the characteristic (002) diffraction peak from 9.5 to 8.1 indicates effective etching of Al layers and formation of the layered MXene structure. According to Bragg's law [50], the interlayer spacing is 1.158 nm, which is notably larger than that of the parent Ti_3_AlC_2_ phase (∼0.9 nm). This increase suggests the removal of Al layers during etching and the subsequent intercalation of water molecules, protons, and surface terminations (─OH, ─F, ═O).

**FIGURE 3:**
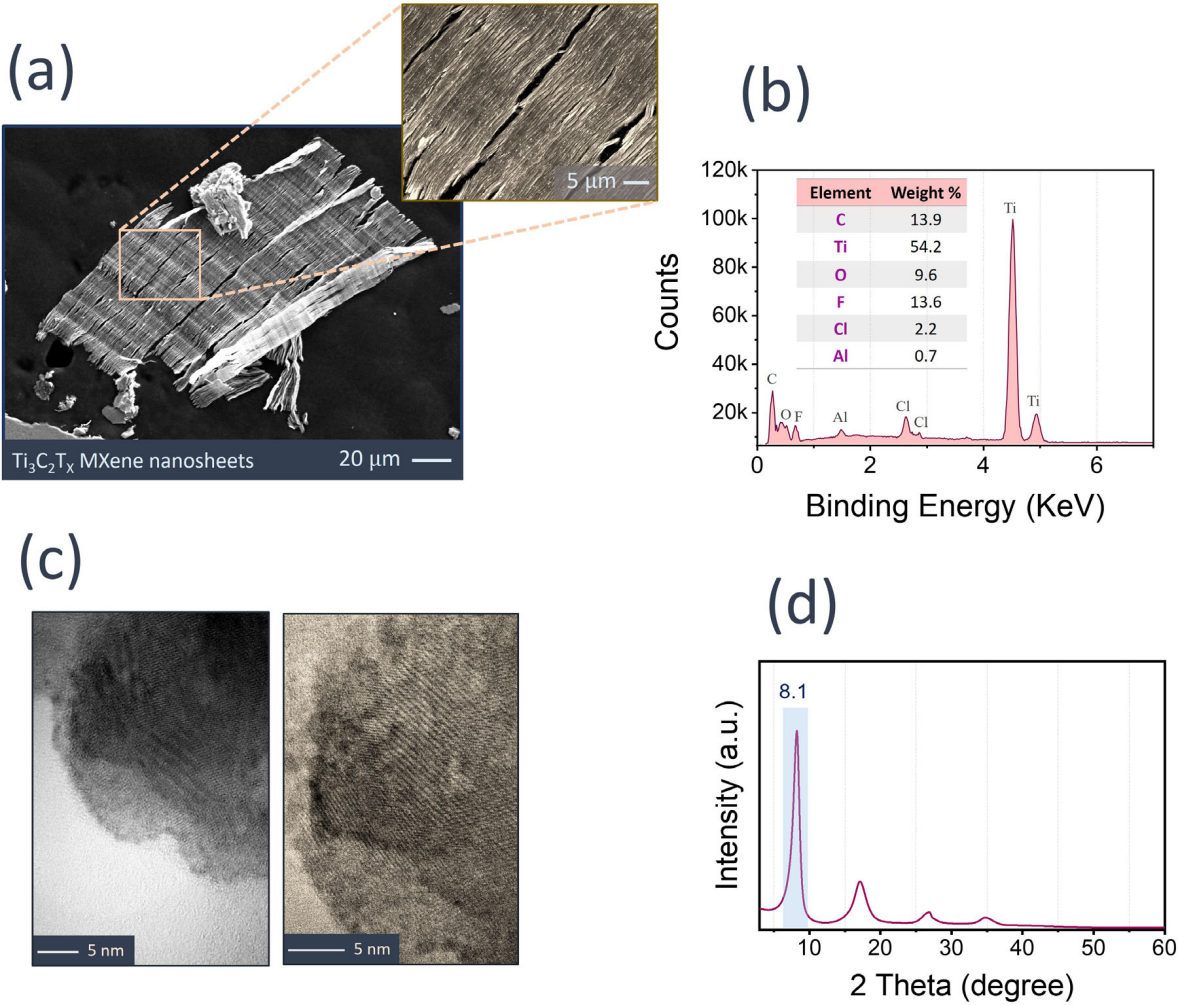
Morphological and chemical characterization of Ti_3_C_2_T*_x_* MXene nanosheets: (a) SEM image, (b) EDAX analysis, (c) HRTEM images showing the crystalline structure of Ti_3_C_2_T*_x_* MXene, and (d) XRD pattern of the MXene powder.

### 3.2 Membrane Characterization

#### 3.2.1 Effect of MXene Loading on Conductivity and FO Performance

To optimize the MXene loading, MX‐T TFC membranes were fabricated with different Ti_3_C_2_T*_x_* concentrations of 5, 15, and 30 mg. The electrical conductivities of the coating layers were measured using a four‐point probe method. Among all membranes, MX‐T 30 exhibited the highest conductivity of 5446.3 S cm^−1^, while the MX‐T 5 and MX‐T 15 membranes showed conductivities of 104.85 and 900.23 S cm^−1^, respectively. The enhanced conductivity of MX‐T 30 arises from the effective overlap of MXene nanosheets, forming a dense, well‐connected conductive network; higher MXene loading reduces interparticle spacing and voids, thereby facilitating electron transport. However, excessive MXene deposition, although beneficial for conductivity, can reduce membrane porosity and increase transport resistance, highlighting a trade‐off between electrical conductivity and water permeability.

To evaluate the effect of MXene loading on the membrane's separation performance, the initial water flux and reverse salt flux were measured in FO mode using 2 M NaCl as the draw solution and DI water as the feed. Table 1 summarizes the corresponding water flux, reverse salt flux, selectivity, and surface conductivity. Incorporation of MXene decreased water permeability but also reduced reverse salt flux, such that the overall selectivity (*J_w_/J_s_*) remained nearly constant across all membranes, with a maximum of 6.37 observed for MX‐T 15. Considering the trade‐off between electrical conductivity, water flux, and selectivity, the MX‐T 15 membrane, with an MXene loading of 0.53 g m^−2^, showed the best performance.

**TABLE 1:** Filtration performance of the TFC‐FO membranes.

Membrane	MXene loading amounts (g m^−2^)	*J_W_* (LMH)	*J_S_* (gMH)	*J*_W_ */J*_S_ (L g^−1^)	Conductivity (S cm^−1^)
TFC	0	15.2	2.81	5.41	—
MX‐T 5 TFC	0.28	13.8	2.64	5.23	104.8 ± 7.2
MX‐T 15 TFC	0.53	11.6	1.82	6.37	900.2± 13.8
MX‐T 30 TFC	1.69	6.1	1.03	6.00	5446.3 ± 19.5

#### 3.2.2 Morphological and Chemical Characterization of Membranes

Figure 4 shows the FESEM and TEM cross‐sectional morphologies of the pristine TFC and MXene‐modified membrane. In Figure 4a, the PA selective layer of the TFC membrane exhibits the characteristic ridge‐and‐valley morphology generated by the heterogeneous diffusion of MPD monomers during interfacial polymerization [49]. The PA layer on the top surface of the MX‐B TFC membrane displayed bigger surface features, likely due to greater diffusion of MPD monomers into the reaction zone. During PA synthesis for MX‐B, the MXene layer was coated on the back side of the PES support, which helped retain more MPD solution within the support, increasing local MPD concentration during polymerization and thereby modifying the PA surface morphology.

**FIGURE 4:**
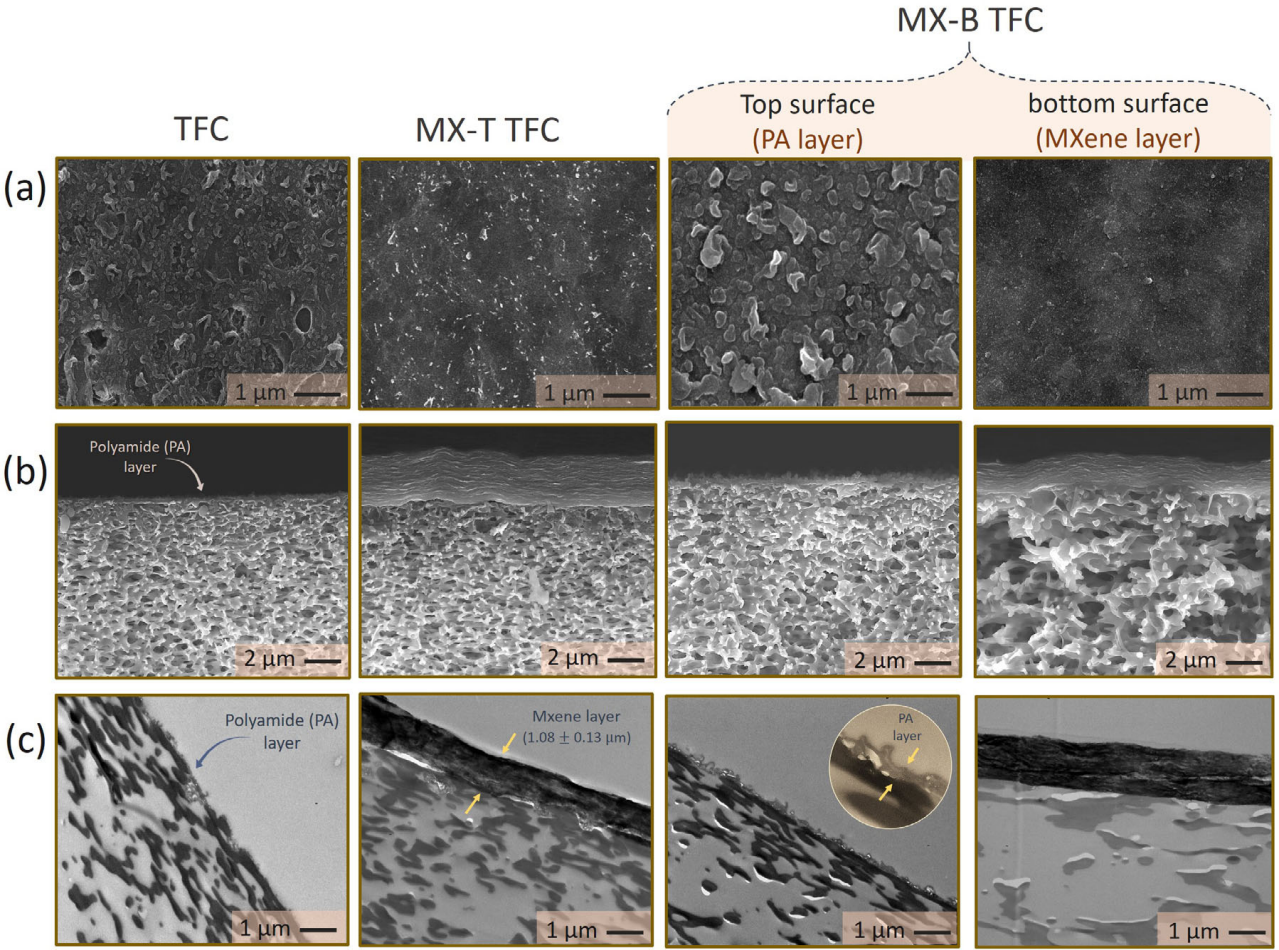
(a) Top‐surface FESEM images, (b) cross‐section FESEM images, and (c) cross‐section TEM images of TFC, MX‐T, and MX‐B membranes. For the MX‐B TFC membrane, both the top surface (PA layer) and bottom surface (MXene layer) were examined to capture the dual‐sided structural features. The PA layer represents the selective skin formed on the top surface, while the MXene layer is the conductive coating on the opposite side.

Top surface FESEM images of the MXene layer in both MX‐T and MX‐B TFC membranes show a continuous film of nanosheets with a wrinkled, wave‐like morphology [51]. The MXene flakes uniformly covered the pores of both the PA layer and the PES substrate, with no visible defects. The cross‐sectional SEM image in Figure 4b reveals a thin, dense PA layer uniformly formed on the porous PES support, with a distinct interface that confirms successful interfacial polymerization and strong adhesion between the selective and support layers. SEM images of MX‐T and the bottom surface of MX‐B show adhesion of the MXene layer to the PA and PVA layers.

Cross‐sectional TEM images of the TFC and MX‐B top surfaces further confirm the formation of a very thin PA layer over the PES surface, with PA penetrating some near‐surface pores. TEM images of the bottom surfaces of MX‐T and MX‐B show a well‐ordered, laminar arrangement of Ti_3_C_2_T*_x_* flakes and strong adhesion of the MXene layer to the PA layer (MX‐T) and the PES substrate (MX‐B). The MXene coating thickness varies locally across the membrane surface, with an average of approximately 1.08 µ*m*.

Figure 5 presents the EDX mapping of the elemental distribution across the cross‐section of the TFC, MX‐T, and MX‐B membranes. The pristine TFC membrane exhibits uniform distributions of carbon (C), oxygen (O), and sulfur (S), corresponding to the PA active layer and PES support. After MXene incorporation, a distinct titanium (Ti) signal appears at the top surface, confirming successful deposition of MXene nanosheets. In both MX‐T and MX‐B membranes, the Ti layer is concentrated near the surface, indicating that MXene forms a thin coating rather than penetrating deeply into the substrate. Overall, the EDX mappings verify the successful fabrication of MXene‐modified composite membranes with well‐defined elemental stratification.

**FIGURE 5:**
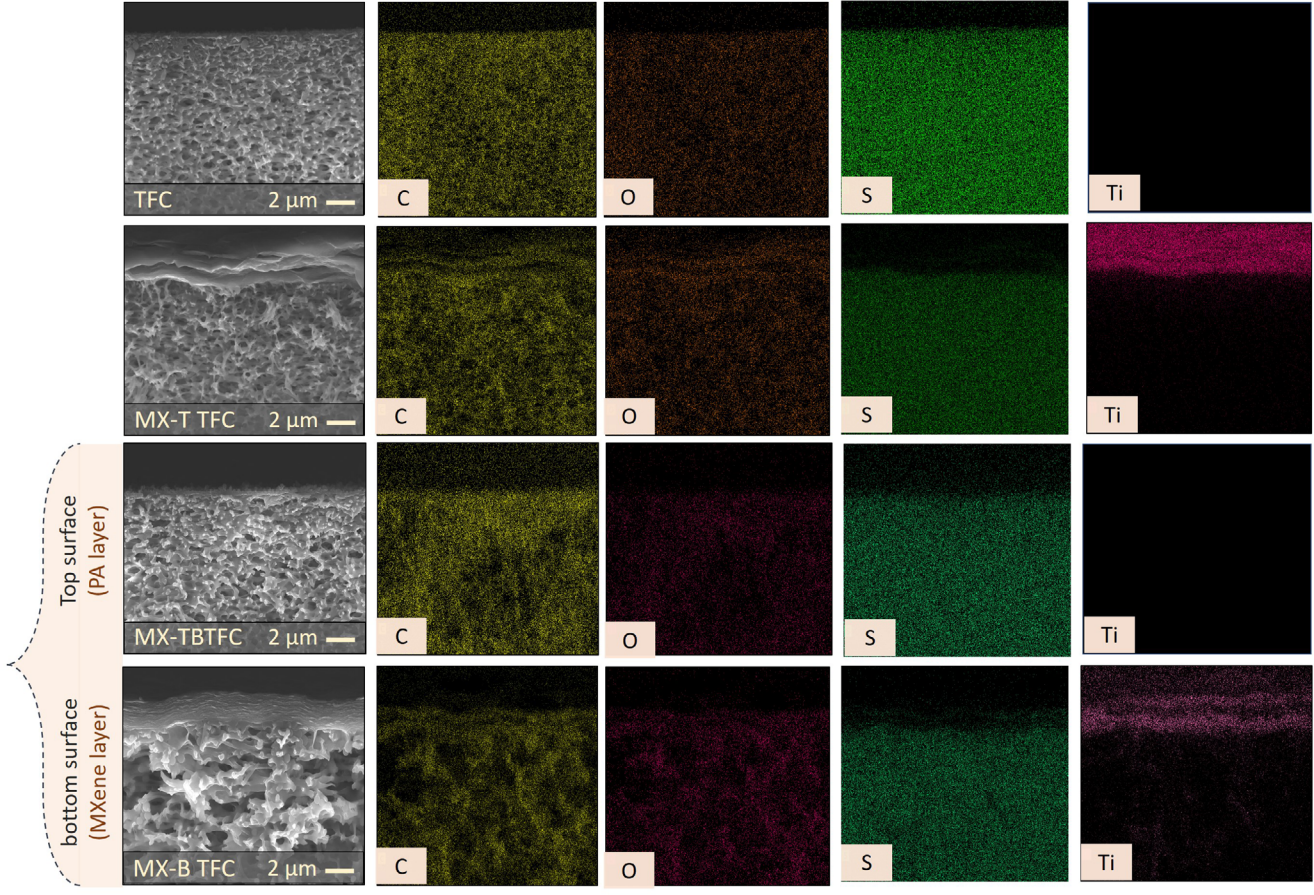
EDX color mappings of C, N, S, and Ti for TFC, MX‐T, and MX‐B membranes.

AFM was employed to assess the effect of MXene coating on surface roughness. Figure 6 shows the 3D and 2D AFM surface topography, surface roughness (average roughness, R_a_, and root‐mean‐square roughness, R_q_), and water contact angles of the membranes. The 3D AFM images show that the TFC membranes have a characteristic ridge‐and‐valley surface structure with an average roughness of 52.97 nm [52]. The top surfaces of TFC and MX‐B membranes have the same roughness, consistent with the presence of a PA layer in both cases.

**FIGURE 6:**
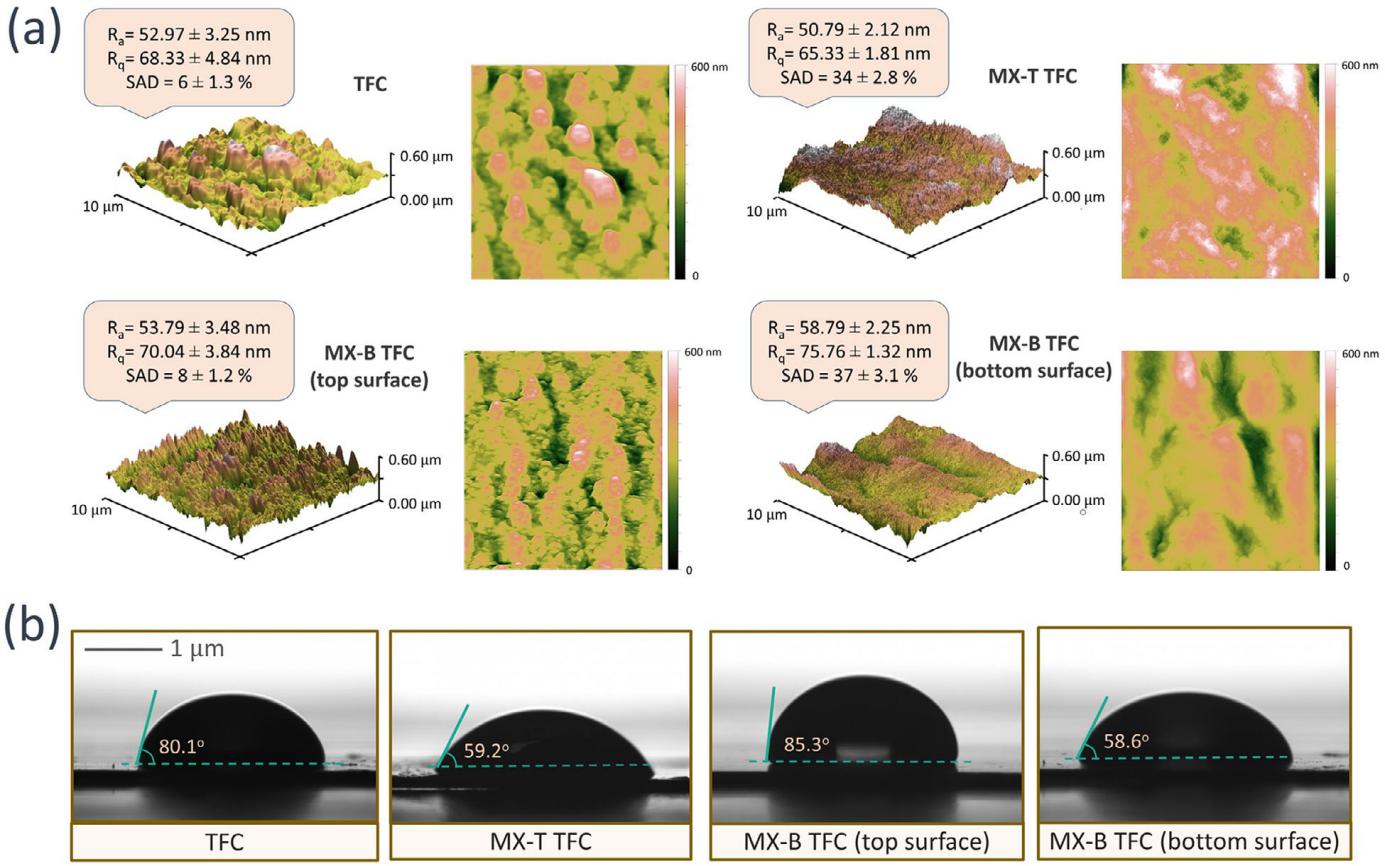
(a) AFM 3D and 2D micrographs and the roughness data, and (b) water contact angle of synthesized TFC membranes.

Coating Ti_3_C_2_T*_x_* on TFC did not considerably alter the surface roughness, where the average roughness of MX‐T and the back surface of MX‐B showed average roughness of 50.79 and 58.79 nm, respectively. The surface area difference (SAD) was calculated as 6% (TFC), 8% (top MX‐B), 34% (MX‐T), and 37% (back MX‐B). SAD quantifies the percentage increase in the actual 3D surface area relative to the projected 3D area. Unlike conventional roughness parameters (R_a_ and R_q_) that reflect only height amplitude, SAD also captures surface slope and curvature. Thus, even when Ra and R_q_ remain nearly constant, a higher SAD indicates the presence of more sloped, wrinkled, or folded surface features. The elevated SAD values after MXene coating confirm that depositing Ti_3_C_2_T_x_ nanosheets introduced additional nanoscale wrinkles and edge structures, increasing the effective surface area without significantly changing vertical roughness.

The water contact angle decreased from 80.1° for the TFC membrane to 59.2° for MX‐T, reflecting the higher hydrophilicity of MXene nanosheets relative to the PA layer due to their abundant oxygen‐containing functional groups. For the MX‐B membrane, the PA‐ and MXene‐coated surfaces exhibited contact angles of 85.3° and 58.6°, respectively, which closely matched those of the TFC and MX‐T membranes. These results demonstrate that incorporating MXene increases surface wettability and enhances the hydrophilicity of TFC membranes.

Figure 7a presents the zeta potential profiles of the pristine TFC and MXene‐coated membranes over a pH range of 4–9. The PA surface is negatively charged across this range, primarily due to the deprotonation of carboxylic groups formed during interfacial polymerization. Upon coating with MXene, the surface charge becomes more negative. This increased negativity arises from the O‐terminated and hydroxyl groups (─O/─OH) on the Ti_3_C_2_T*_x_* surface, which partially deprotonate to ─O^−^ under these conditions, increasing the density of negatively charged sites on the membrane.

**FIGURE 7:**
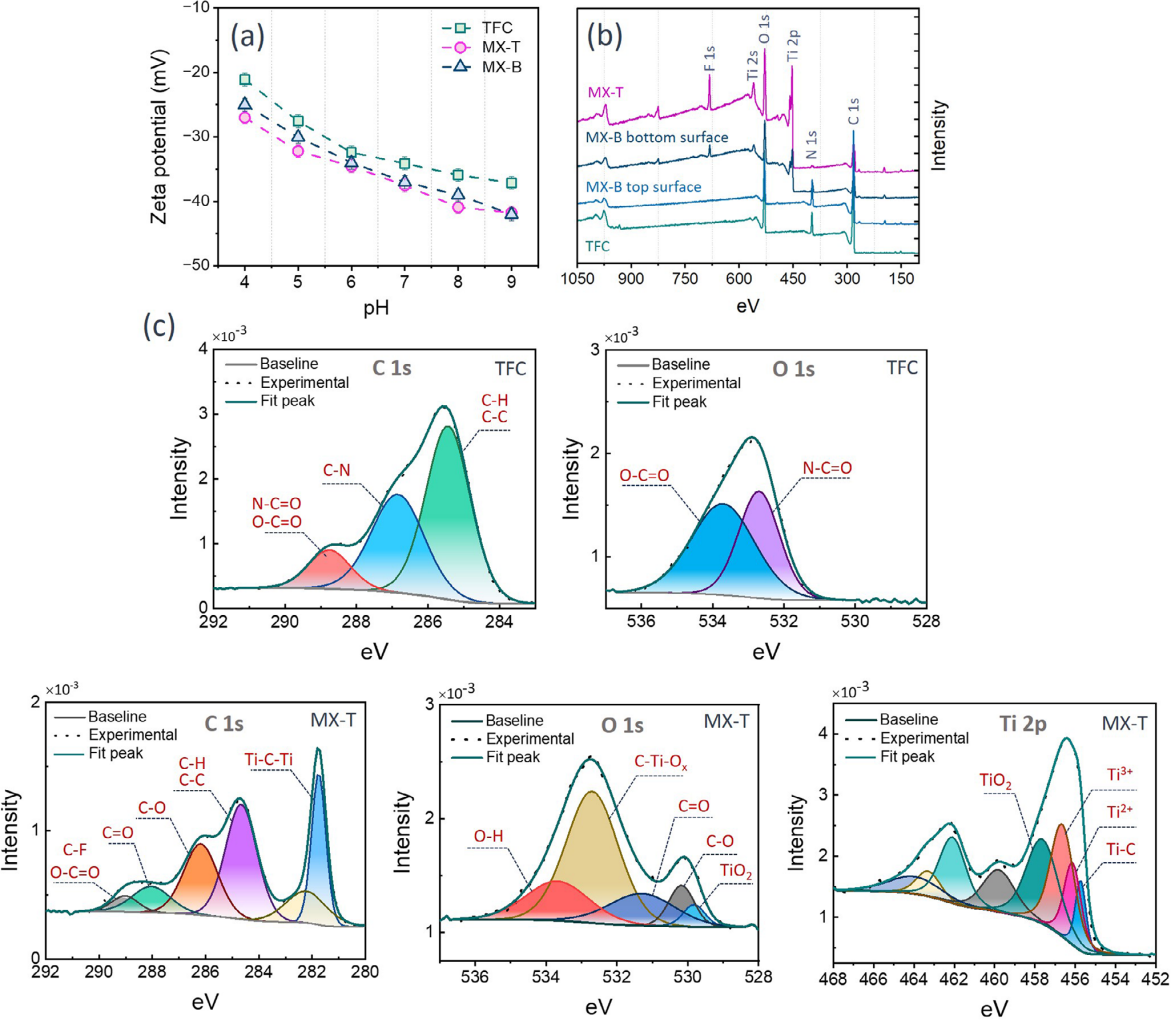
(a) Variation of zeta potential as a function of pH for TFC, MX‐T, and MX‐B membranes, (b) XPS general survey of TFC, MX‐T, MX‐B top surface, and MX‐B, and (c) C 1s, O 1s, and Ti 2p high‐resolution XPS spectra of TFC and MX‐T. Ti 2p spectrum in TFC is absent because there are no MXene nanosheets in the pristine TFC membrane.

Figure 7b presents the XPS general survey spectra of the TFC, MX‐T, and MX‐B membranes. The TFC membrane displays characteristic C 1s (∼285 eV), O 1s (∼531 eV), and N 1s (∼400 eV) peaks, which are consistent with the chemical structure of the polyamide selective layer. In comparison, the survey spectra of the MX‐T and MX‐B bottom surfaces show additional Ti 2p (∼460 eV) and F 1s (∼685 eV) peaks, confirming the presence of titanium carbide and fluorine‐containing surface terminations characteristic of Ti_3_C_2_T*_x_* MXene. These MXene‐coated surfaces also retain the carbon and oxygen signals observed in TFC. By contrast, the top surface of the MX‐B membrane exhibits a spectrum closely resembling that of the TFC membrane.

The high‐resolution XPS spectra in Figure 7c further illustrates the chemical structures of the TFC and MX‐T membranes. For TFC, the C 1s spectrum is deconvoluted to C─C and C─H bonds at 284.3 eV, C─N at 285 eV, and carboxyl and amide functional groups (N─C═O and O─C═O) at 288.9. The corresponding O 1s spectrum is deconvoluted to O─C═O and N─C═O groups, consistent with the chemistry of the aromatic polyamide. For MX‐T, the C 1s spectrum becomes more complex due to contributions from MXene. A prominent peak at approximately 281.5 eV corresponds to the C─Ti bond, which is characteristic of MXene carbide structures. Additional peaks at approximately 284.4 and 287.3 eV originate from graphitic C─C/C─H and C─O bonds, while smaller peaks at 288.0 and 289.2 eV correspond to carbonyl and carboxyl functional groups commonly associated with surface terminations and adsorbed species. The Ti 2p region of MX‐T reveals multiple species, with peaks assigned to Ti‐C, Ti(II), Ti(III), and TiO_2_. The O 1s spectrum similarly shows a diverse range of oxygen species, including TiO_2_, C─O, C═O, C─Ti─O/C─Ti─OH, and O─H species, reflecting the heterogeneous nature of MXene surface terminations. The high‐resolution spectra of MX‐B are provided in the Supporting Information, where the MX‐B top surface resembles the TFC membrane. In contrast, the MX‐B bottom surface closely matches the MX‐T spectrum, further confirming that MXene is present only on the intended side of the membrane.

### 3.3 Electrochemical Characterization of MXene‐Based Membranes

To better understand the electron‐transfer behavior and surface conductivity of the fabricated membranes, electrochemical impedance spectroscopy (EIS), linear sweep voltammetry (LSV), and chronoamperometry (CA) measurements were performed using MX‐B and MX‐T membranes under different electrolyte conditions. These electrochemical analyses provide insight into the interfacial charge‐transfer resistance, electrical contact efficiency, and oxygen reduction activity of the MXene‐coated surfaces, which are critical parameters in anodic oxidation processes.

Figure 8a,b shows the Nyquist plots for the MX‐B and MX‐T membranes in 50 and 200 mM Na_2_SO_4_ solution. For the MX‐B membrane, the ohmic resistance obtained from the high‐frequency intercept was 1.90 Ω in 50 mM Na_2_SO_4_ and 1.10 Ω in 200 mM Na_2_SO_4_ (Figure 8a). This decrease is attributed to the electrolyte's enhanced ionic conductivity at higher salt concentrations. At 200 mM Na_2_SO_4_, both MX‐B and MX‐T membranes exhibited comparable ohmic resistance values of 1.90 and 1.94 Ω, respectively (Figure 8b). However, the Nyquist plot of the MX‐T membrane displayed a smaller semicircle, indicating a lower charge‐transfer resistance (R_ct_) and more efficient interfacial electron transport. This difference likely arises from variations in the microstructure and surface morphology of the MXene layer deposited on the TFC substrate.

**FIGURE 8:**
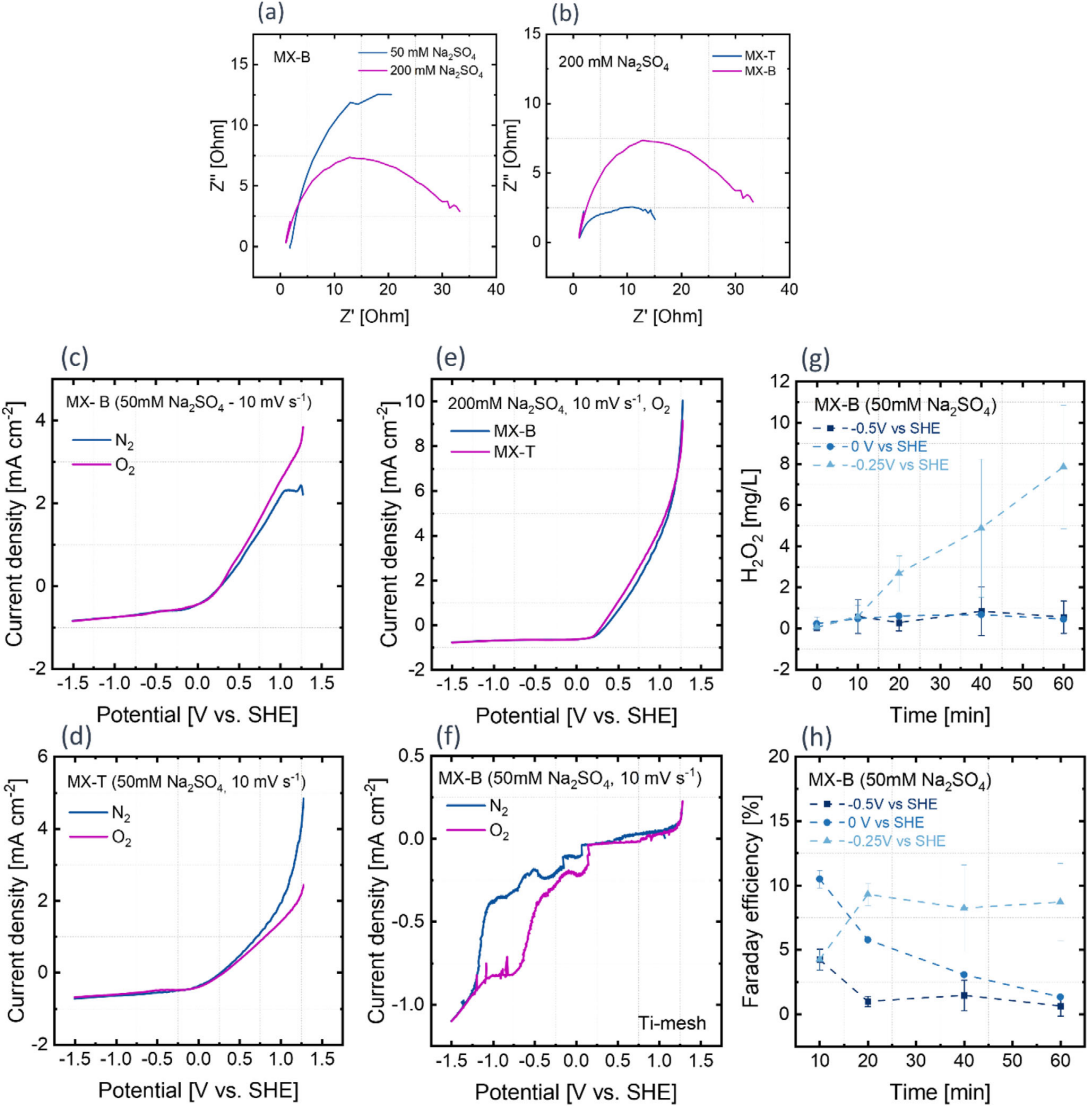
Electrochemical impedance spectroscopy (EIS) results: Nyquist plots for MXene‐based membranes of (a) MX‐B membrane measured in 50 and 200 mM Na_2_SO_4_ electrolytes, and (b) MX‐B and MX‐T membranes at 200 mM Na_2_SO_4_ electrolyte. Linear sweep voltammetry (LSV) and constant potential (chronoamperometry, CA) results: LSV curves of (c) MX‐B membrane in 50 mM Na_2_SO_4_ under N_2_‐ and O_2_‐saturated conditions, (d) MX‐T membrane in 50 mM Na_2_SO_4_ under N_2_‐ and O_2_‐saturated conditions, (e) MX‐T and MX‐B membranes in 200 mM Na_2_SO_4_ under O_2_ saturation, (f) MX‐B membrane in 50 mM Na_2_SO_4_ with Ti‐mesh as a current collector under N_2_‐ and O_2_‐saturated conditions, (g) H_2_O_2_ concentration in constant potential experiments as a function of time at 0, −0.25, and −0.5 V vs. SHE, and (h) corresponding Faradaic efficiency (FE). The results show that H_2_O_2_ production is most pronounced at −0.25 V, whereas activity is negligible at 0 and −0.5 V vs. SHE.

Figure 8c,d presents the linear sweep voltammetry (LSV) curves of both membranes, recorded at a scan rate of 10 mV s^−1^ in 50 mM Na_2_SO_4_ under N_2−_ and O_2_‐saturated conditions. In both cases, the current response shows a minor difference between the two gases, indicating limited oxygen‐reduction reaction (ORR) activity under the experimental conditions. Increasing the Na_2_SO_4_ concentration to 200 mM reduces the ohmic resistance, as demonstrated in the EIS data (Figure 8a,b). However, this improved conductivity yields only a modest increase in current response, particularly in the negative potential range where oxygen reduction is expected.

Furthermore, both membranes display similar electrochemical behavior under these conditions (Figure 8e). A notable distinction between the two membranes arises in their short‐term electrochemical stability. MX‐B maintains consistent electrochemical performance even after several repeated EIS and LSV measurements, whereas MX‐T shows progressive deterioration of the MXene coating. In another configuration, a Ti‐mesh was placed directly on top of the MX‐B as a current collector, improving contact across the electrode area. As shown in Figure 8f, this configuration enhances the current response with a clear distinction between N_2_ and O_2_ saturation, particularly from +0.2 to −1.0 V vs. SHE. In addition, several broad redox features are observed in the negative potential region of Figure 8f and are attributed to reversible surface faradaic processes on the Ti‐mesh/MXene interface. These features are likely associated with the reduction and oxidation of native titanium oxide species on the Ti mesh, as well as proton‐coupled electron transfer involving oxygen‐containing surface terminations of MXene (e.g., ═O and ─OH groups). The broader, less‐defined shapes of these peaks suggest pseudocapacitive behavior rather than discrete bulk redox transitions. Under O_2−_ saturated conditions, the cathodic current becomes more pronounced due to the contribution of ORR, which partially overlaps with the intrinsic surface redox processes. The enhanced separation between the N_2_ and O_2−_ saturated curves, therefore, confirms improved electrochemical accessibility and ORR activity when the Ti‐mesh current collector is employed. Based on this improved setup, three representative potentials (0 V, −0.25 V, and −0.5 V vs. SHE) were selected for subsequent constant‐potential experiments to evaluate the membranes' capability for the two‐electron oxygen reduction reaction (2e^−^ ORR) toward H_2_O_2_ generation.

To evaluate the electrochemical mechanisms more comprehensively, we also investigated the membrane's cathodic activity, specifically its potential to generate hydrogen peroxide (H_2_O_2_) via the oxygen reduction reaction (ORR). Although the MXene membrane functions primarily as the anode in the hybrid PFAS degradation process, testing its cathodic behavior at negative potentials is a critical control. By quantifying H_2_O_2_ production, we aimed to determine whether cathodic pathways could contribute to the overall treatment or whether degradation is strictly driven by anodic oxidation.

The performance of the MX‐B membrane under constant potential operation was evaluated at three selected cathodic potentials (0 V, −0.25 V, and −0.5 V vs. SHE) in 50 mM Na_2_SO_4_ (Figure 8). At both 0 V and −0.5 V, the concentration of H_2_O_2_ remained close to zero throughout the measurement period, indicating a negligible contribution from the two‐electron oxygen reduction pathway. However, at −0.25 V, H_2_O_2_ accumulation was observed, reaching up to 7.8 mg L^−^^1^ after 60 min (Figure 8g), confirming that the MXene‐based membrane exhibits some catalytic activity toward the two‐electron ORR at this specific potential.

Despite this measurable H_2_O_2_ production, the Faradaic efficiency (FE) remained relatively low across all tested potentials, with a maximum value of ∼10% at −0.25 V vs. SHE (Figure 8h). Under the tested conditions, H_2_O_2_ formation on the conductive membrane is minimal. This confirms that electro‐generated H_2_O_2_ plays a negligible role in the actual treatment process. By effectively ruling out this cathodic pathway, these control experiments support the conclusion that PFOA degradation in the coupled system is overwhelmingly driven by anodic mechanisms, namely, direct electron transfer and the generation of reactive oxygen species (e.g., OH) at the electroactive membrane interface.

To evaluate the physical stability of the coated layer, a dynamic leaching test was performed by monitoring titanium release from the modified membranes under continuous aqueous agitation. Membrane specimens were immersed in deionized water and stirred at 100 rpm for 21 days. Daily aliquots were collected and analyzed for dissolved Ti to quantify any leaching from the Ti_3_C_2_ MXene coating. Throughout the test, Ti concentrations remained below the analytical detection limit, indicating negligible leaching. Using Ti release as an indicator for coating stability, these results demonstrate the high stability of the Ti_3_C_2_ MXene layer under prolonged aqueous stirring.

While the MX‐T configuration showed progressive coating deterioration during repeated electrochemical cycling, the MX‐B design eliminated this vulnerability. The PVA binding layer anchored the MXene nanosheets to the porous support, effectively preventing physical detachment. Combined with the lack of titanium leaching over the 21‐day continuous agitation test, the consistent EIS and LSV performance confirms that the MX‐B membrane maintains both structural integrity and catalytic activity. This robust interfacial adhesion enables the membrane to withstand prolonged operation and high current densities without failure under severe oxidative conditions.

### 3.4 Transport Performance of Synthesized TFC Membranes

Figure 9a,b presents the water flux of TFC, MX‐T, and MX‐B membranes as a function of draw solution concentrations in two configurations, AL‐FS and AL‐DS. In all cases, water flux increased with draw solution concentration from 0.5 to 2.0 M NaCl because the larger osmotic pressure difference provides a stronger driving force for water transport across the active layer. Comparing Figure 9a,b, the water flux of the pristine TFC membrane is higher in the AL‐DS configuration than in the AL‐FS mode. In AL‐DS mode, the active layer faces the draw solution, so water first passes through the porous support and then through the selective layer before entering the draw solution. This orientation helps reduce ICP inside the support layer because the draw solution directly contacts the active surface, maintaining a stronger osmotic driving force. As a result, the effective osmotic pressure difference across the membrane is higher, leading to greater water permeability compared to the AL‐FS mode.

**FIGURE 9:**
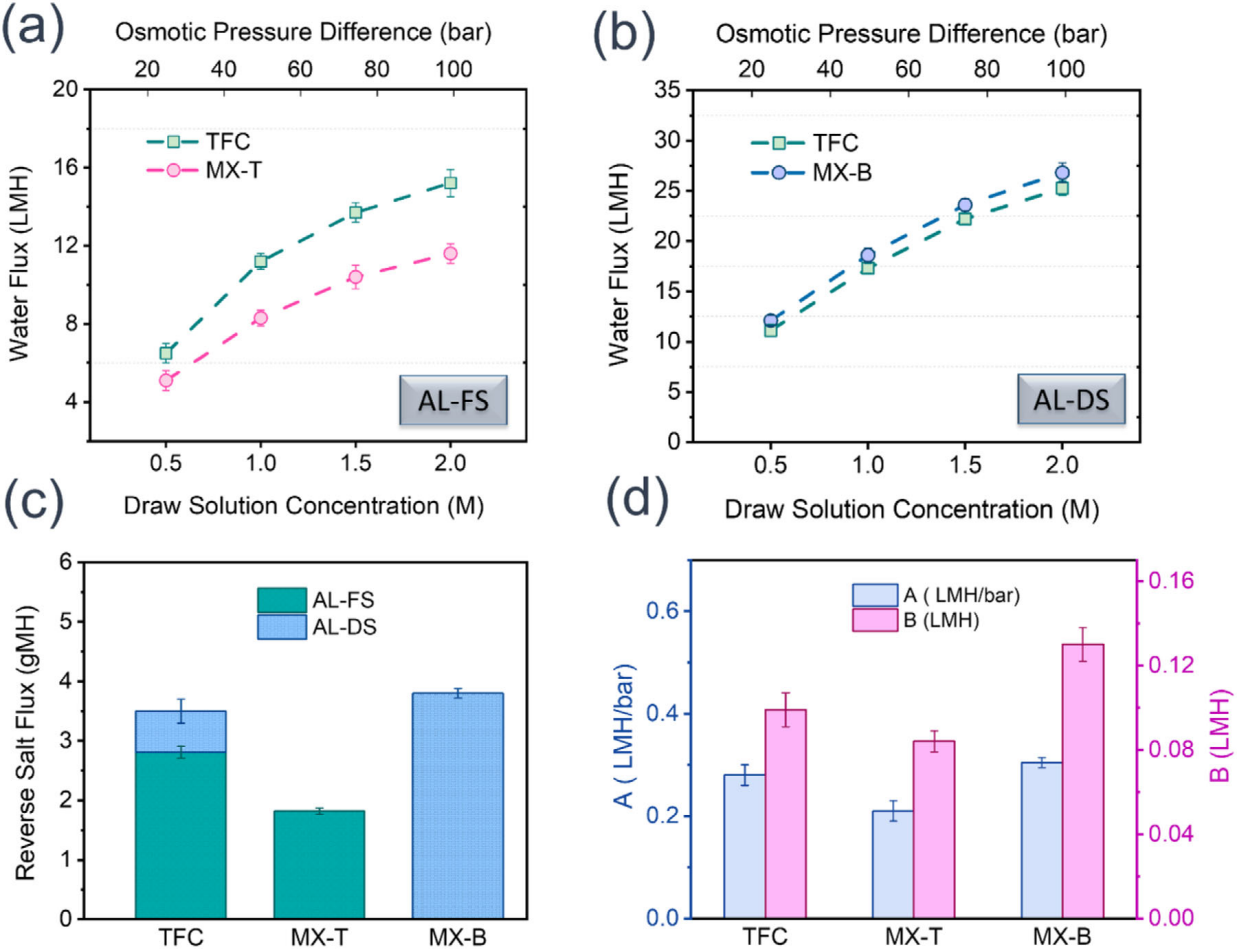
(a) Water flux of TFC and MX‐T in the AL‐FS configuration, (b) water flux of TFC and MX‐B in the AL‐DS configuration, (c) reverse salt flux of TFC, MX‐T, and MX‐B, and (d) transport parameters (A and B) of TFC, MX‐T, and MX‐B membranes. Error bars indicate the standard deviation (SD) obtained from three independent experiments. The water flux values shown in panels (a) and (b) correspond to the initial flux at the start of filtration, and the reverse salt flux was determined based on the increase in NaCl concentration in the feed solution over a 2‐h filtration period.

As shown in Figure 9a, the TFC membrane exhibited higher flux than the MX‐T membrane. The slightly reduced performance of the MX‐T membrane is attributed to the added MXene coating, which introduces additional mass‐transfer resistance. The nonlinear increase in flux with concentration also suggests the growing influence of ICP, in which solutes accumulate within the porous PES support layer in the AL‐FS configuration, thereby reducing the effective osmotic pressure gradient across the membranes [53]. Figure 9b demonstrates that the pristine TFC and MX‐B membranes had almost similar water flux in the AL‐DS configuration.

Figure 9c compares the reverse salt flux of the TFC, MX‐T, and MX‐B membranes tested with a 2 M NaCl draw solution. The MXene coating on the PA surface of the MX‐T membrane introduced additional transport resistance, reducing reverse salt diffusion compared with the pristine TFC membrane. This decrease can be attributed to the formation of a denser and more negatively charged surface that limited ion passage through the selective layer. In contrast, the MX‐B membrane exhibited a slightly higher reverse salt flux than the TFC membrane in the AL‐DS configuration.

Figure 9d presents the transport parameters (A and B) of the TFC, MX‐T, and MX‐B membranes. The water permeability coefficient (A) follows the same trend as the water flux results, with MX‐B showing the highest value due to its improved surface hydrophilicity and reduced structural resistance. The MX‐T membrane, on the other hand, exhibits a slightly lower A value compared to the pristine TFC membrane.

In the MX‐B configuration, the MXene‐PVA layer is deposited beneath the PES substrate, thereby altering the hydrophilicity and pore wetting of the PES before IP. The presence of this MXene layer at the back of the PES helps retain the MPD solution within the substrate for a longer time. As a result, the local MPD monomer concentration at the organic‐aqueous interface is higher, leading to faster reaction kinetics but a lower cross‐linking density in the resulting polyamide layer. This less cross‐linked (and slightly looser) structure enhances water permeability but also allows greater passage of dissolved ions, explaining the higher A and B values of MX‐B.

### 3.5 PFAS Removal of Synthesized TFC Membranes

A series of FO experiments was performed to evaluate the water flux and PFOA rejection of the fabricated TFC membranes. Each experiment was conducted over 4 h using 2 M NaCl as the draw solution and a feed solution of 20 mg L^─1^ in water. Figure 10a shows that the TFC and MX‐T membranes exhibited initial water fluxes of 15.2 and 11.6 LMH, respectively, in AL‐FS. The water fluxes then declined by about 40% and 35%, reaching final fluxes of 9.0 and 6.8 LMH. This decline is primarily attributed to the dilution of the draw solution, which reduces the osmotic driving force. Both membranes exhibited excellent PFOA rejection of approximately 99%. Figure 10b shows that, in the AL‐DS orientation, the TFC and MX‐B membranes demonstrated initial water fluxes of 28.5 and 26.1 LMH, respectively. These fluxes decreased by 34% and 31% over the 4‐h filtration period, reaching final values of 18.6 and 17.7 LMH, respectively. The TFC membrane exhibited PFOA rejection ranging from 99.43% to 99.68%, while the MX‐T and MX‐B membranes achieved higher rejection values ranging from 99.72% to 99.94%. These high rejection values demonstrate the excellent barrier properties of the polyamide selective layer toward PFOA transport.

**FIGURE 10:**
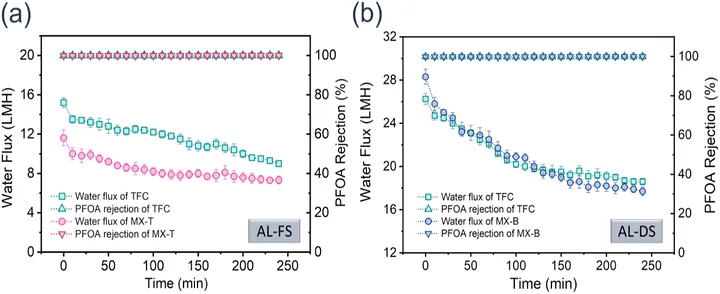
(a) Water flux and PFOA rejection of TFC and MX‐T membranes in the AL‐FS mode, and (b) TFC and MX‐B membranes in the AL‐DS mode.

Therefore, the integration of in‐situ anodic oxidation provides a critical enhancement to the FO separation process. While the dense polyamide layer effectively rejects PFOA, the electroconductive MXene layer simultaneously serves as an anode, generating reactive oxidative species that degrade PFOA accumulated near the membrane interface. By continuously oxidizing concentrated PFOA within the boundary layer, the system mitigates severe concentration polarization and suppresses interfacial solute accumulation. This synergistic coupling of membrane separation and electrochemical degradation not only helps sustain the osmotic driving force and maintain water flux but also minimizes the long‐term risk of contaminant passage.

The main mechanisms for PFOA rejection in TFC‐based FO membranes are size exclusion and electrostatic repulsion between the negatively charged membrane surface and anionic PFOA molecules. As water molecules selectively permeate the membrane, the PFOA concentration at the membrane surface increases, thereby increasing concentration polarization. This phenomenon can further contribute to reducing the permeation.

### 3.6 Degradation of PFOA by Combining Oxidation and Osmotic Dewatering

In AO, the degradation rate of PFOA is predominantly controlled by the applied current density, which dictates both the rate of direct electron transfer and the generation of reactive oxidants. The process begins with direct electron transfer of PFOA molecules to the anode surface, thereby enabling their decomposition [35]. As the current density increases, the direct oxidation of PFOA is enhanced due to a higher rate of electron exchange between the anode and PFOA species [54]. Additionally, elevated current densities promote the formation of hydroxyl radicals (•OH) via water discharge at the anode surface [55].

Figure 11 illustrates the effect of current density on total organic carbon (TOC) removal using a titanium cathode and an MXene‐coated membrane as the anode. At different current densities, the initial TOC concentration (4.64 mg/L) decreased sharply within the first hour of operation, then declined more slowly. When only the AO process was applied, TOC removal increased from 29% to 36% as the current density rose from 200 to 350 A/m^2^. Further increasing the current density to 500 A/m^2^ increased TOC removal to 50%, likely due to oxygen evolution reactions (OER) competing with PFOA oxidation at higher current densities [56].

**FIGURE 11:**
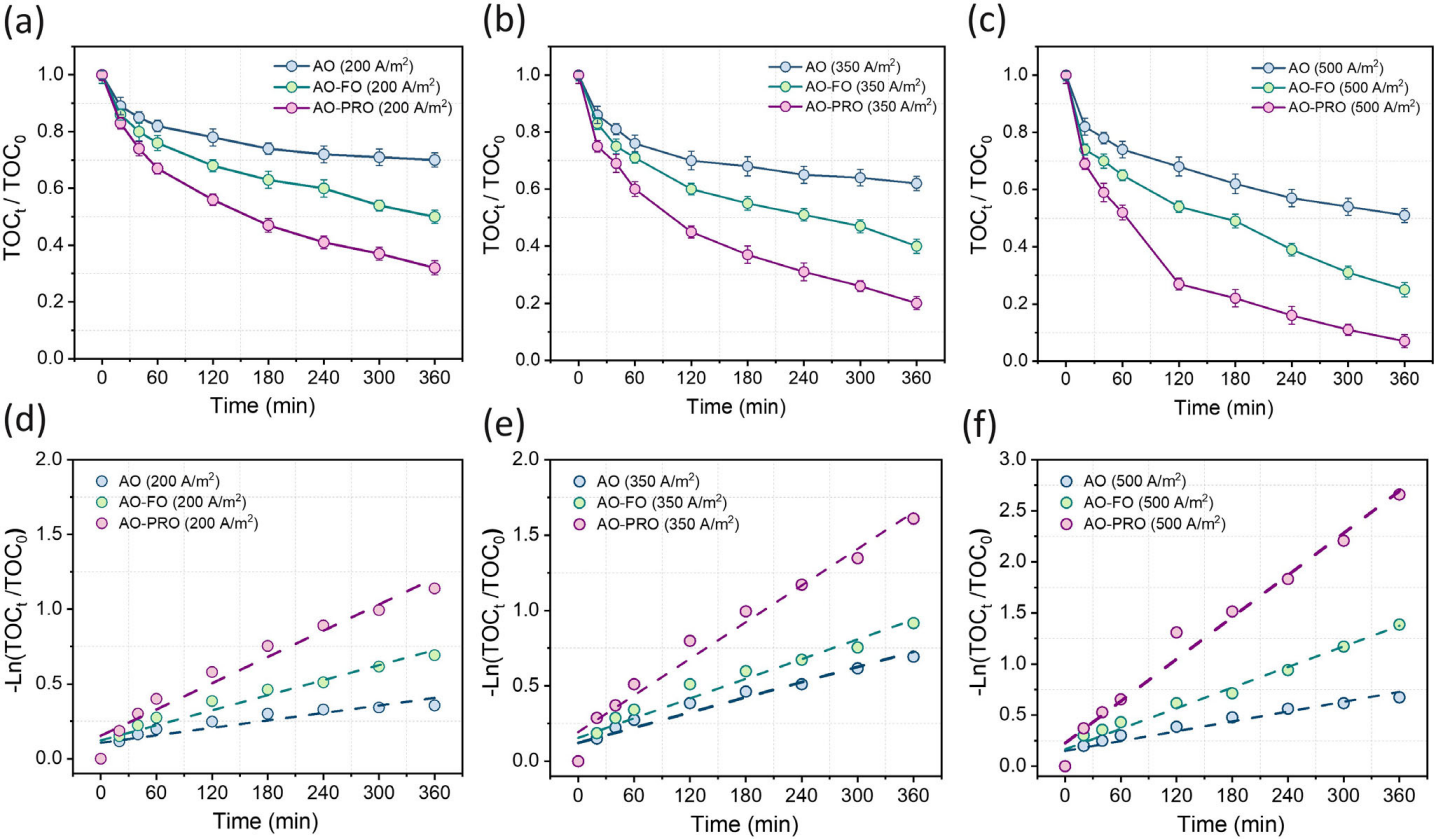
(a–c) Comparing PFOA mineralization for AO (no dewatering), AO‐FO, and AO‐PRO at current densities of 200, 350, and 500 A/m^2^, and (d–f) the corresponding pseudo‐first‐order kinetic fits from TOC decay. Feed solution: 20 mg/L PFOA, 0.05 M Na_2_SO_4_.

Integrating osmotic dewatering (FO and PRO modes) with AO enhances degradation kinetics by continuously concentrating solutes in the electrolyte solution. This effect is more pronounced in the AO‐PRO than in the AO‐FO, owing to the higher water flux achieved in PRO mode. Figure 11 shows that the AO‐PRO consistently achieved higher TOC removal than the AO‐FO and AO. At 200 A/m^2^, TOC removal reached 29% in AO but increased to 51% in the AO‐FO and 69% in the AO‐PRO. Increasing the current density to 500 A/m^2^ further intensified TOC removal, achieving 50%, 75%, and 93% removal for AO, AO‐FO, and AO‐PRO, respectively. These results demonstrate that continuous dewatering of the feed solution significantly improves pollutant concentration and oxidation efficiency, accelerating overall mineralization in the hybrid system [35]. While continuous dewatering in PRO mode provides a stronger driving force for contaminant concentration, it is important to note that the AO‐PRO configuration uses the MX‐B membrane, which has lower charge‐transfer resistance and greater physical stability than the MX‐T membrane used in AO‐FO. Therefore, the exceptional 93% TOC removal achieved in AO‐PRO at 500 A/m^2^ results from enhanced osmotic dewatering in PRO mode and the superior electrochemical and mass‐transfer properties of the MX‐B architecture.

It is worth noting that the key performance outcome of this research is not only PFAS rejection, although the membranes achieved approximately 99% PFOA rejection, but also the ability of the coupled system to mineralize concentrated PFOA at the membrane interface. At 500 A/m^2^, the AO‐FO system reached 75% TOC removal, while the AO‐PRO system reached 93% TOC removal. In contrast, standalone AO showed substantially lower mineralization under comparable conditions. This comparison demonstrates that the osmotic dewatering step is not a passive addition; it directly improves electrochemical degradation by increasing local PFOA concentration and reducing mass‐transfer limitations.

Kinetic analysis revealed that TOC decay followed pseudo‐first‐order reaction kinetics (Figure 11d–f), with the corresponding apparent rate constants (K_app_) summarized in Table 2. Among all tested conditions, AO at 200 A/m^2^ and AO‐PRO at 500 A/m^2^ exhibited the lowest (0.048 h^─1^) and highest (0.474 h^─1^) K_app_ values, respectively, confirming the positive effect of osmotic dewatering on degradation kinetics. This enhancement is attributed to concentrating organic solutes near the anode surface, thereby accelerating oxidation reactions that would otherwise proceed slowly under dilute conditions.

**TABLE 2:** Apparent pseudo‐first‐order kinetic constants (K_app_) and correlation coefficients.

Test	TOC decay K _app_ (h^─1^) R^2^	
AO 200	0.048	0.9296
AO‐FO 200	0.102	0.9598
AO‐PRO 200	0.176	0.9725
AO 350	0.106	0.9412
AO‐FO 350	0.132	0.9668
AO‐PRO 350	0.232	0.9886
AO 500	0.146	0.9474
AO‐FO 500	0.204	0.9811
AO‐PRO 500	0.474	0.9879

Figure 12 shows that the complete degradation of 20 mg/L PFOA was attained within 6 h across the current densities. Notably, the AO‐PRO system exhibited faster removal rates compared to AO and AO‐FO processes. However, TOC data revealed the persistence of organic residues even after full PFOA removal, indicating the formation of shorter‐chain intermediates [57].

**FIGURE 12:**
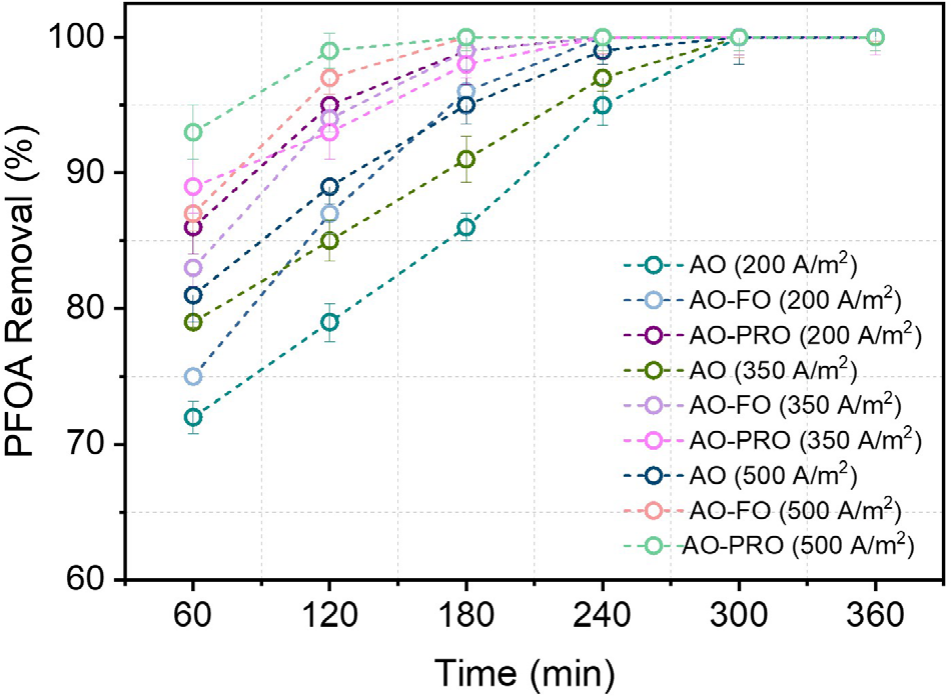
Effect of dewatering on PFOA removal during the AO process, both alone and hybrid process (AO‐FO and AO‐PRO) in different current densities. Experimental conditions: 0.05 M Na_2_SO_4_ supporting electrolyte.

These findings demonstrate that integrating osmotic dewatering with AO markedly reduces overall treatment time and improves process efficiency, potentially reducing operational energy demand. In this study, we achieved a higher apparent degradation rate constant than in our previous work using the hybrid process of AO, electro‐Fenton, and membrane distillation [35]. Adsorption‐based approaches, such as granular activated carbon (GAC), can retain PFAS without inducing degradation and often show variable removal efficiencies, particularly for short‐chain species. Furthermore, such methods require post‐treatment processes to manage and treat PFAS‐laden regeneration solutions effectively [58, 59]. Similarly, nanofiltration (NF) and reverse osmosis (RO) can achieve >90% PFAS removal but generate concentrated waste streams requiring additional treatment [16]. In contrast, the hybrid AO and osmotic dewatering process achieves high mineralization with minimal secondary.

### 3.7 Products of PFOA Degradation

The main organic decomposition intermediates of PFOA were identified in full‐scan mode using LC‐Triple Quadrupole Mass Spectrometry (LC‐Triple‐Quad‐MS) at different stages of the hybrid AO‐PRO process at a current density of 350 A/m^2^. Compound identification was performed based on the m/z (mass‐to‐charge ratio) of parent ions (precursor ions) and daughter ions (product ions). Quantification was based on the chromatographic peak areas of individual analytes.

Figure 13 shows 6 short‐chain perfluorocarboxylic acids (PFCAs), including C_6_F_13_COOH (PFHpA), C_5_F_11_COOH (PFHxA), C_4_F_9_COOH (PFPeA), C_3_F_7_COOH (PFBA), C_2_F_5_COOH (PFPrA), and CF_3_COOH (TFA). The decomposition mechanism involved continuous elimination of a CF_2_ unit, as reported in our previous study [35]. Figure 13a presents the temporal evolution of short‐chain PFCA concentrations during PFOA degradation. The longer‐chain intermediates, such as PFHpA (C7) and PFHxA (C6), reached their maximum concentrations at 20 and 30 min, respectively, and then decreased thereafter. The concentrations of the remaining intermediates (C1‐C5) increased after 1 h and then decreased. This trend demonstrated that the longer‐chain intermediates appeared at the start of the reaction and then decomposed into shorter‐chain products. The low residual PFCA levels confirm the high oxidative efficiency of the hybrid AO‐PRO process. Table 3 shows the concentration of short‐chain intermediates after 6 h. While our integrated AO‐PRO system serves as a highly efficient primary degradation technology that eliminates most long‐chain PFAS, a final polishing step (such as highly selective ion exchange or granular activated carbon) would likely be required in a full‐scale treatment train. While the >99% degradation efficiency of the AO‐PRO system alone may be sufficient to achieve ng/L regulatory targets for typical municipal wastewater with trace baseline concentrations, this subsequent polishing step ensures absolute compliance with stringent global regulatory guidelines (e.g., Canada's 30 ng/L total PFAS target) when treating industrial waters with heavily elevated initial PFAS loads.

**FIGURE 13:**
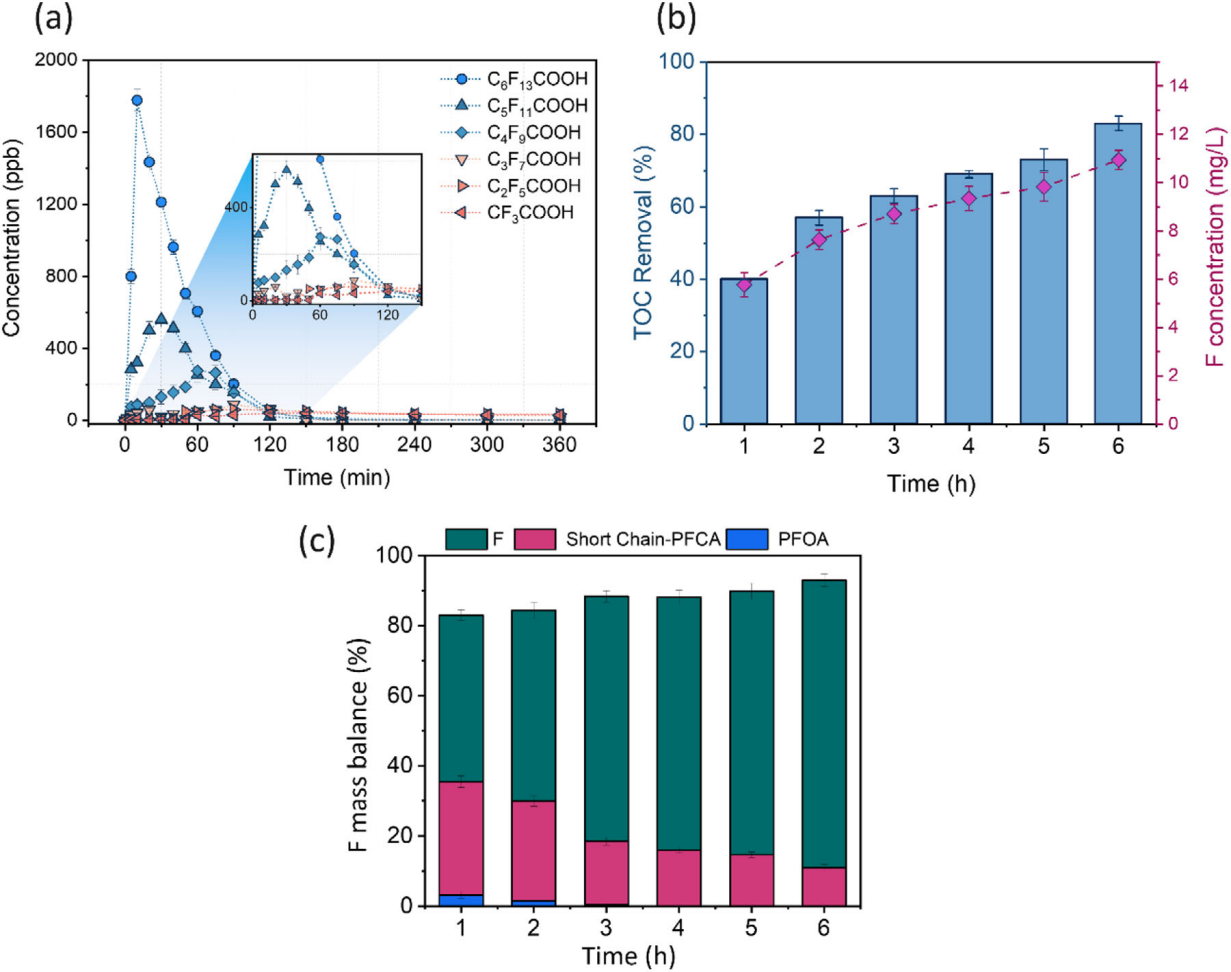
(a) Variation of short‐chain perfluorinated compound concentrations with reaction time, (b) fluoride ion concentration and TOC removal during PFOA degradation, and (c) Fluorine mass balance during PFOA degradation. Error bars indicate the standard deviation (SD) obtained from three independent experiments.

**TABLE 3:** Concentration of remaining short‐chain intermediates after 6 h.

PFAS Type	Concentration (ppb)
C_4_F_9_COOH	2.03
C_3_F_7_COOH	14.4
C_2_F_5_COOH	38.2
CF_3_COOH	53.6

The TOC removal profile (Figure 13b) demonstrates the mineralization of intermediates over extended operation. The gradual decline in organic carbon content corresponds to the successive breakdown of perfluorinated chains into shorter carboxylic acids and ultimately CO_2_, indicating continuous oxidation. Fluoride ion (F^−^) generation during PFOA degradation was quantified via ion chromatography (IC) to determine the defluorination ratio. As shown in Figure 13b, the F^−^ concentration increased steadily over time, reaching 10.84 mg/L after 6 h of treatment, corresponding to an 80% defluorination efficiency. This result confirms the effective cleavage of C─F bonds by heterogeneous •OH radicals generated at the anode. Given that PFOA (C_8_HF_15_O_2_, 414.07 g mol^−^^1^) contains a fluorine mass fraction of 0.688, a 20 mg/L PFOA solution corresponds to 13.76 mg/L of total fluorine. Considering an 80% degradation efficiency, the theoretical fluoride concentration of 11 mg/L is in close agreement with the measured value [60]. To verify the completeness of this degradation pathway, the total fluorine recovery rate was evaluated using the formula: (n_F_^−^ + n_F, short‐chain intermediates_ + n_F, undegraded PFOA_)/n_F, total_. As illustrated in Figure 13c, this mass balance shows a clear shift from organically bound fluorine to mineralized fluoride over time. Early in the reaction, most fluorine is tied up in the parent PFOA and the immediate C6/C7 breakdown products. As anodic oxidation progresses, this organic fluorine fraction shrinks rapidly while the free F^−^ fraction dominates. Ultimately, this high overall fluorine recovery confirms that our analysis effectively identified and tracked the major degradation intermediates of PFOA.

To quantitatively contextualize the performance of the proposed AO‐PRO and AO‐FO configurations, we benchmarked our findings against state‐of‐the‐art electroconductive and hybrid PFAS degradation technologies (Table 4). A direct comparison with the MXene electrosorption/oxidation system reported by Ma et al. (2023) demonstrates that while their system achieved rapid parent compound decay (>99% PFOA removal at +6 V) via surface‐charge attraction, our AO‐PRO system leverages draw‐driven osmotic concentration to achieve 93% TOC mineralization at 500 A/m^2^ without requiring hydraulic pressure. Furthermore, comparing the AO‐PRO system to our previously reported MD‐electrochemical system (95% TOC removal at 500 A/m^2^) highlights a critical operational trade‐off. Although MD achieved a slightly higher mineralization rate, it required continuous thermal input to vaporize feed water (60°C). The current AO‐PRO layout achieves a comparable 93% mineralization rate isothermally at ambient temperatures (22°C), substantially reducing operational energy demands.

**TABLE 4:** Quantitative comparison of hybrid process for PFAS degradation.

Hybrid process	Material and design	Process mechanism	Target contaminant	Operating conditions	PFAS removal (%)	Mineralization (%)	Defluorination (%)	Key features
Osmotic concentration + anodic oxidation (This work, AO‐PRO)	Ti_3_C_2_T_*x*_ MXene on porous support layer (MX‐B)	Pressure retarded osmosis (PRO) draw‐driven dewatering + in situ anodic oxidation	PFOA (10 mg L^−1^)	500 A/m^2^, 50 mM Na_2_SO_4_, 6 h, 22°C (Isothermal)	100%	93%	∼82%	Draw‐driven concentration enriches local PFAS at the anode without hydraulic pressure or thermal energy input
Osmotic concentration + Anodic oxidation (This work, AO‐FO)	Ti_3_C_2_T_*x*_ MXene on active layer (MX‐B)	Forward osmosis (FO) draw‐driven dewatering + In‐situ anodic oxidation	PFOA (10 mg L^−1^)	500 A/m^2^, 50 mM Na_2_SO_4_, 6 h, 22°C (Isothermal)	100%	75%	∼41%	Draw‐driven concentration enriches local PFAS at the anode without hydraulic pressure or thermal energy input
Membrane distillation + Electro‐Fenton/Oxidation [35]	PTFE membrane	Membrane Distillation (MD) thermal concentration + Cathodic electro‐Fenton + BDD anodic oxidation	PFOA (20 mg L^−1^)	500 A/m^2^, Feed Temp: 60°C, 6 h	>98%	95%	∼88%	Excellent mineralization; requires thermal energy to supply the latent heat of vaporization for feed distillation
Electro sorption + Anodic Oxidation [44]	Oxygen‐terminated Ti_3_C_2_T_*x*_ MXene membrane	Cross‐flow hydraulic filtration + Applied electric field attraction (Electrosorption) + Anodic oxidation	PFOA (1 mg L^−1^) PFBA (1 mg L^−1^)	+ 6 V vs. Ag/AgCl, 0.1 M Na_2_SO_4_, 3 h	>99%	*N.A*.	∼67%	Electrosorptive enrichment (215.9 mg g^−1^) enhances surface collision; requires applied hydraulic cross‐flow pressure
Adsorption / Ion Exchange + Electrochemical Oxidation [61]	Anion Exchange Resin (AER)/GAC bed coupled with BDD Anode	Pre‐concentration on high‐capacity sorbent + Periodic electrochemical desorption and anodic destruction	PFOS (50 µg L^−1^) PFOA (50 µg L^−1^)	Regeneration current density: 200 A/m^2^, brine electrolyte	>95%	∼81%	∼75%	Minimizes continuous power consumption by decoupling high‐volume capture from low‐volume batch destruction.
NF/RO retentate concentration + BDD oxidation [62]	Polyamide NF270 membrane + BDD electrochemical Cell	Commercial nanofiltration (NF) retention + Batch electrochemical oxidation of concentrated retentate stream	PFOA (100 µg L^−1^)	NF pressure: 5 bar; BDD current density: 300 A/m^2^	>99%	∼85%	∼79%	High overall water recovery; retentate matrix complexity (scaling ions) can passivate the anode over extended runs.

To further contextualize the performance of the proposed AO‐PRO and AO‐FO systems, Table 4 provides a quantitative comparison with recently reported electro‐membrane processes for PFAS degradation, detailing membrane materials, operational modes, target contaminants, and mineralization efficiencies.

### 3.8 Specific Energy Consumption Analysis

When evaluating the system's overall energy footprint, it is important to separate the demands of the membrane process from the electrochemical treatment. Because the FO and PRO modes operate via natural osmotic pressure gradients, the standalone separation step demands very little energy, limited almost entirely to running the low‐pressure circulation pumps. As a result, the DC power supply driving anodic oxidation (AO) accounts for nearly all of the system's specific energy consumption (SEC).

Figure 14 presents this SEC calculated over three operating intervals (0–2, 2–4, and 4–6 h) for the AO, AO‐FO, and AO‐PRO configurations. During the first two hours of operation at 200 A/m^2^, the standalone AO process required 12.11 kWh of electrical energy to remove 1 g of TOC. Under the same operating conditions, coupling AO with FO and PRO substantially reduced this energy demand, yielding SEC values of 6.25 and 2.36 kWh per gram of TOC removed for AO‐FO and AO‐PRO, respectively.

**FIGURE 14:**
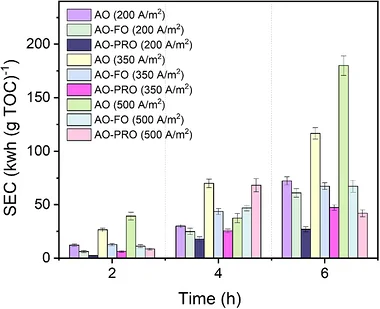
Specific energy consumption (SEC) calculated from TOC removal results during 6 h of operation. Experimental conditions for the feed solution were [PFOA]_0_ = 20 ppm, [Na_2_SO_4_] = 0.05 M.

The data also reveal a strong relationship between electrochemical energy consumption and changes in PFOA concentration over time. As Figure 14 illustrates, the SEC jumps noticeably during the later operating intervals (2–4 h and 4–6 h) across all configurations. As shown in Figure 11, TOC decreases sharply during the first two hours; this relatively high initial PFOA concentration ensures that the generated hydroxyl radicals readily find and react with organic molecules, keeping the initial SEC low. However, as the treatment effectively destroys the target organics, the remaining solution becomes increasingly dilute. The system continues to draw electrical power to generate oxidants, but far fewer target molecules remain to oxidize. At lower target concentrations, these reactions become less likely, slowing the mineralization kinetics. The process essentially loses some of its kinetic efficiency, causing the energy required to remove each remaining gram of TOC to rise steadily as treatment progresses.

Although increasing the applied current density further increased the overall SEC, this temporal trend remained consistent across all tested conditions. Comparing the cumulative SEC over the full 6‐h period confirms that integrating osmotic dewatering through AO‐PRO and AO‐FO.

### 3.9 Comparative Assessment of PFOS Removal and Degradation

Figure 15a,b depicts the osmotic dewatering performance of the TFC, MX‐B, and MX‐T membranes using PFOS solution as the feed. Similar to the results obtained for PFOA, all membranes exhibited excellent PFOS rejection during the experiments, maintaining rejection values above 99%. The high PFOS rejection can be attributed to the dense polyamide selective layer, which effectively restricts PFAS transport through a combination of size exclusion and electrostatic repulsion. Since both PFOA and PFOS are long‐chain anionic PFAS with comparable molecular dimensions and hydrated sizes, the transport mechanisms governing their rejection are expected to be similar. Incorporating MXene into the membrane structure did not compromise selectivity, confirming the stability of the selective layer during prolonged osmotic dewatering operation.

**FIGURE 15:**
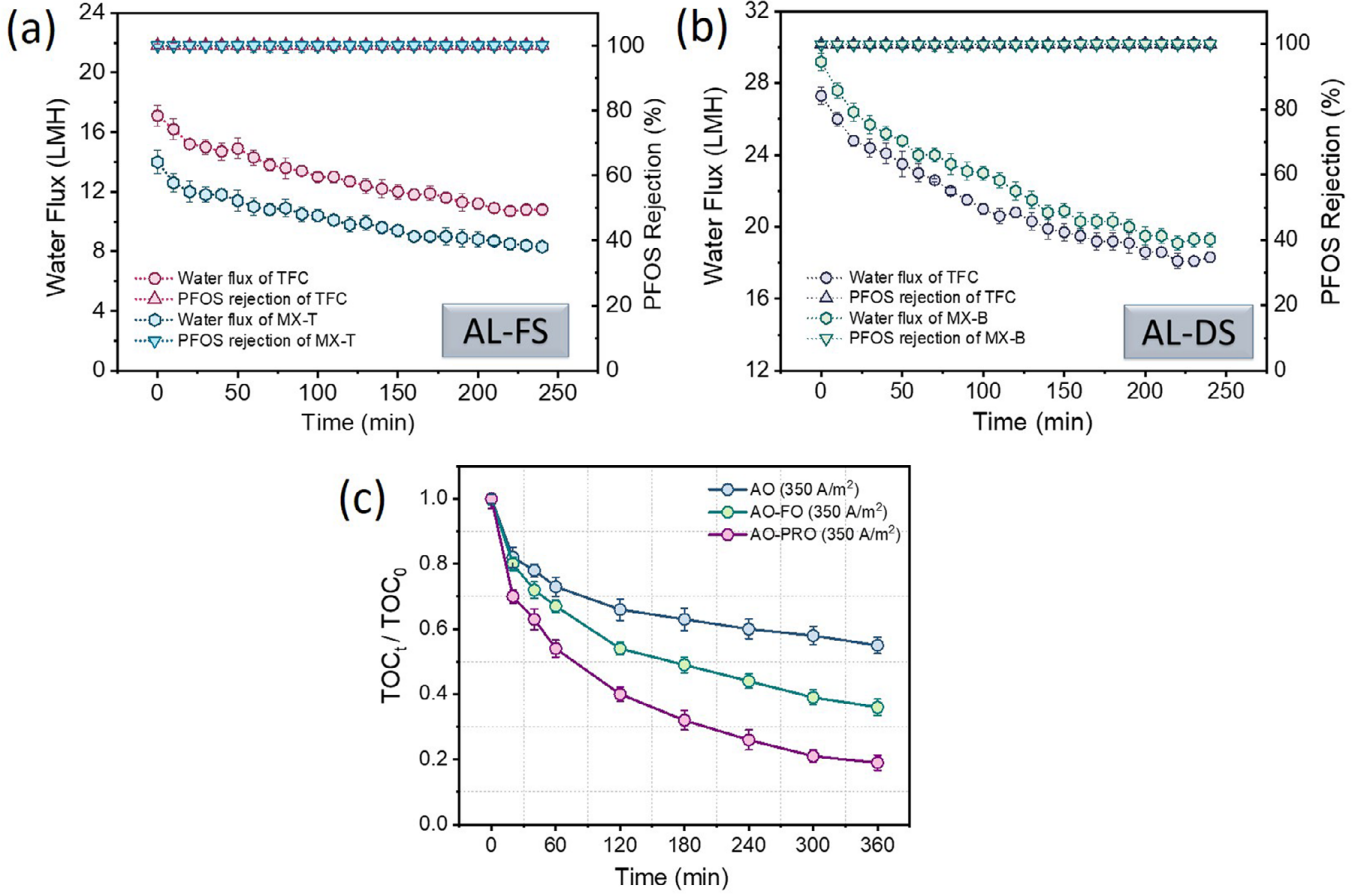
(a) PFOS rejection and water flux performance of the TFC and MX‐T membranes in AL‐FS configuration, (b) PFOS rejection and water flux performance of the TFC and MX‐B membranes in AL‐DS configuration, and (c) PFOS mineralization performance under AO, AO‐FO, and AO‐PRO configurations at a current density of 350 A/m^2^.

Figure 15c shows the normalized TOC concentration (TOC_t_/TOC_0_) during PFOS degradation under AO, AO‐FO, and AO‐PRO configurations at a current density of 350 A/m^2^. All configurations showed a continuous decrease in TOC, confirming effective PFOS mineralization. Among the tested systems, AO‐PRO achieved the highest degradation performance, followed by AO‐FO and conventional AO. The enhanced performance of the integrated systems can be attributed to the continuous water extraction and concentration effects provided by the osmotic process, which improved the transport of PFOS toward the electroactive MXene surface and increased contact between PFOS molecules and reactive oxidizing species.

Comparing PFOS results with those for PFOA showed that PFOS degraded slightly more efficiently under identical operating conditions. The primary reason is PFOS's greater affinity for the conductive MXene surface. Because of the sulfonate functional group and its greater hydrophobicity, PFOS shows stronger adsorption and interfacial interactions with conductive surfaces through a combination of hydrophobic and electrostatic interactions. This promotes PFOS accumulation near the reactive MXene surface, where direct electron transfer and radical‐mediated oxidation occur. Consequently, PFOS can undergo more effective surface‐mediated degradation pathways, including desulfonation and subsequent mineralization reactions. In addition, although PFOA also degraded effectively, complete mineralization was more challenging because PFOA degradation generates shorter‐chain perfluorocarboxylic acid intermediates that are often more persistent and require additional reaction time for further degradation. As a result, residual organic carbon remained for a longer period during PFOA treatment. PFOS's stronger adsorption behavior therefore facilitates its electrochemical oxidation and contributes to the slightly lower TOC values observed in the PFOS experiments.

## 4 Conclusion

This study demonstrates a novel treatment strategy that couples AO with osmotic dewatering to enhance PFOA removal and mineralization. The integrated AO‐FO and AO‐PRO configurations were designed to overcome the inherently slow electrochemical oxidation kinetics associated with low contaminant concentrations. In these systems, PFOA undergoes anodic oxidation on an electrically conductive MXene‐coated membrane, while water is simultaneously withdrawn from the reaction cell by an osmotic pressure gradient across a TFC membrane. The MXene layer therefore serves as both an electrode and a membrane component involved in separation. PFOA degradation kinetics improved markedly as the applied current density was increased from 200 to 500 A/m^2^. At 500 A/m^2^, the AO‐PRO system achieved complete PFOA removal and substantial mineralization, as indicated by a 93% reduction in TOC, whereas the standalone AO processachieved only ∼50% removal under comparable conditions. These results demonstrate the beneficial role of osmotic dewatering in enhancing electrochemcial oxidation. The improved performance of the AO‐PRO system may also be associated with the favorable water‐transport properties of the MXene layer in the MX‐B membrane. Consistent with these findings, the AO‐FO configuration exhibited TOC decay reaction rate constants approximately three to four times higher than those of AO alone, depending on the applied current density. Mechanistic analysis indicated that PFOA degradation proceeded predominantly via direct electron transfer at the electroconductive MXene membrane surface, with hydroxyl‐radical‐mediated oxidation providing an additional synergistic contribution.

Synthetic solutions were used to establish a well‐defined baseline, isolate the degradation mechanisms, and track intermediate breakdown products without background consituents. Transition of this technology to real‐world applications will require evaluation using authentic wastewater matrices. Complex effluents naturally contain background organic matter, radical scavengers such as carbonate species, and competing ions that may consume reactive species, promote membrane fouling, and alter PFOA degradation kinetics. Future studies should therefore evaluate the coupled AO‐FO and AO‐PRO systems using real industrial or municipal wastewaters. Such studies will be critical for assessing the long‐term resistance of the membranes to to fouling and optimizing specific energy consumption under practical, scaled‐up conditions. Although the MX‐B configuration demonstrated stable electrochemical performance under the conditions investigated, as supported by a 21‐day agitation test, repeated EIS/LSV measurements, and titanium ion release analysis, its long‐term durability under continuous anodic operation at high current densities requires comprehensive evaluation.

Overall, the coupled AO‐FO and AO‐PRO systems provide a promising, energy‐efficient technology for PFOA removal from contaminated waters. Their ability to achieve rapid and extensive PFOA degradation while reducing specific electrical energy consumption underscores their potential for scale‐up and practical implementation in contaminated‐water treatment.

## Conflicts of Interest

The authors declare no conflicts of interest.

## Supporting information




**Supporting File**: smll75540‐sup‐0001‐SuppMat.docx.

## Data Availability

The data that support the findings of this study are available from the corresponding author upon reasonable request.
