# Supplementary material for: Electroconductive MXene‐Thin‐Film‐Composite Membranes for Coupled Osmotic Concentration and PFAS Mineralization
Source: Small. 2026 Aug 31;22(61):e75540. doi: 10.1002/smll.75540 (PMC13725401; doi:10.1002/smll.75540)
Supplement: Supplementary file 1 [file SMLL-22-e75540-s001.docx]

Supporting Information

Electroconductive MXene-TFC membranes for coupled osmotic concentration and PFAS mineralization

Afrouz Yousefi^1,2^, Pooria Karami^1^, Vahid Rad^3^, Amir Aghaei^1^, Mojtaba Mohseni^5^, Lingling Yang^2^, Masoud Soroush^3,4^, Matthias Wessling^5^, Mohamed Gamal El-Din^2*^, Mohtada Sadrzadeh^1*^

^1^ Department of Mechanical Engineering, 10-241 Donadeo Innovation Center for Engineering, Advanced Water Research Lab (AWRL), University of Alberta, Edmonton, AB T6G 1H9, Canada

^2^ Department of Civil and Environmental Engineering, University of Alberta, Edmonton, Alberta T6G 1H9, Canada

^3^Department of Chemical and Biological Engineering, Drexel University, Philadelphia 19104, PA, USA

^4^Department of Materials Science and Engineering, Drexel University, Philadelphia, 19104, PA, USA

^5^RWTH Aachen University, Aachener Verfahrenstechnik - Chemical Process Engineering, Forckenbeckstr. 51, 52074, Aachen, Germany

* Corresponding authors: [sadrzade@ualberta.ca](mailto:sadrzade@ualberta.ca); [mgg1@ualberta.ca](mailto:mgg1@ualberta.ca)

**S1. MXene and Membrane Characterization**

X-ray diffraction (XRD) spectra of the MXene powder were recorded by a Rigaku XRD Ultima IV machine at a rate of 2^o^/min. Membrane surface wettability was evaluated by water contact angle (WCA) analysis. A 2 mL droplet of DI water in air was used in the sessile drop measurement technique, employing a contact angle analyzer (Kruss DSA 100 GmbH, Germany). The water droplet was placed on the membrane's surface using a micro syringe. Three or more droplets were placed on the surface for each sample, and the average contact angle was subsequently determined.

Membrane surface and cross-section morphology were examined by field emission scanning electron microscopy (FESEM, Zeiss Sigma 300 VP) at an acceleration voltage of 15 kV. The samples were first immersed in liquid nitrogen and then fractured carefully for cross-sectional FESEM imaging. Additionally, the elemental composition of the membranes was determined using energy-dispersive X-ray spectroscopy (EDS). The thickness of the polyamide (PA) selective layer was determined from cross-sectional transmission electron microscopy (TEM) images (Philips/FEI Morgagni 268, Netherlands). Prior to imaging, membranes were stained with uranyl acetate and lead citrate, embedded in Spurr’s resin, and sectioned into ultrathin slices using an ultramicrotome (Reichert-Jung Ultracut E, USA). The sections were mounted on 300-mesh copper grids and observed under TEM at 80 kV.

The membrane surface roughness was measured using tapping-mode atomic force microscopy (AFM, Dimension ICON, Bruker, Germany). Measurements were performed using a silicon nitride probe, scanning a 10 µm × 10 µm area at 1 Hz. The collected data were analyzed using NanoScope Analysis software (Version 1.40r3, Bruker, Germany), and the root-mean-square roughness (*R*_a_) was reported for each sample.

The Surpass™ 3 Electrokinetic analyzer (Anton Paar, Graz, Austria) was used to assess the zeta potential of the membranes. To measure zeta potential over the pH range 4-9, a 1 mM KCl solution was used, and pH was adjusted with NaOH and HCL. The elemental composition and chemical bonding states of the TFC membranes were characterized using a Kratos AXIS ULTRA XPS system equipped with a monochromatic Al Kα X-ray source.

Sheet resistivity was measured using a four-point probe (PRO4-4000) connected to a source meter (Keithley 2601A). For each membrane, 10 readings were recorded at various locations to ensure accurate measurements. The electrical conductivity (S/cm) of the membrane was subsequently determined using the equation: Conductivity = 1/Resistivity.

All electrochemical characterization experiments were conducted in a flow-through module (Flex-E-Cell, FXC Engineering GmbH) as shown in **Figure S1A** (bottom left). The cell was operated in a three-electrode configuration, with a 4.5 × 5.0 cm² platinum-coated expanded titanium sheet (Magneto) serving as the counter electrode (CE), and two pieces of 1.5 × 3 cm² as-synthesized MXene-coated poly (vinyl alcohol) (PVA) layers on a PES support (Type A) or MXene-coated polyamide thin-film composite (TFC) membranes (Type B) serving as the working electrodes (WE). A potassium-saturated Hg_2_SO_4_ electrode (0.64 V vs. SHE) was used as the reference electrode (RE). A 100 mL solution of 50 mM or 200 mM Na_2_SO_4_ was circulated through the module at 50 mL/min. The electrolyte was continuously bubbled with either N₂ or O₂ for 20 minutes prior to and during testing to ensure saturation.

Electrochemical impedance spectroscopy (EIS) was first performed to establish the baseline impedance of the electrochemical cell (**Figure S1A**). A small alternating current (AC) perturbation was applied at the open-circuit potential, and the resulting voltage response was recorded over the frequency range 0.1 Hz to 100 kHz.

Subsequently, linear sweep voltammetry (LSV) experiments were conducted in the same flow-through module (**Figure S1A**) with an identical electrode configuration and electrolyte flow rate. The potential was swept between 1.25 V and –1.5 V vs. SHE to evaluate membrane electrochemical activity. For constant potential (chronoamperometry, CA) experiments, a divided cell configuration was employed (**Figure S1B**). A Nafion 212 cation exchange membrane was introduced as a separator and proton conductor. The catholyte consisted of 50 mM Na_2_SO_4_, while 500 mM H_2_SO_4_ was used as the anolyte. Initial experiments with 100 mL of catholyte showed negligible H_2_O_2_ production (<0.5 mg/L); therefore, the catholyte volume was reduced to 50 mL to increase the H_2_O_2_ concentration and improve measurement accuracy.

**Figure S1.** Schematic of the experimental setup in a flow-through module: (A) EIS and LSV experiments, (B) CA experiments.

**S2. XRD Characterization of Ti₃C₂Tₓ MXene and MX-T Membrane**

The XRD patterns of the parent Ti_3_AlC_2_ MAX phase and the synthesized Ti_3_C_2_T_X_ MXene are compared in **Figure S2**. The characteristic diffraction peaks of the Ti_3_AlC_2_ MAX phase were substantially reduced or disappeared after etching, while the characteristic (002) reflection shifted from approximately 9.5^°^ for the MAX phase to 8.1^°^ for Ti_3_C_2_T_x_ MXene. According to Bragg's law, this shift corresponds to an increase in interlayer spacing from approximately 0.90 nm to 1.16 nm, confirming successful removal of the Al layers during etching and formation of the layered MXene structure. Similar XRD characteristics, including a downward shift of the (002) peak, an increase in d-spacing, and the disappearance of characteristic MAX-phase reflections, have been widely reported as definitive evidence of the successful conversion of Ti_3_AlC_2_ into Ti_3_C_2_T_x_ MXene.

**Figure S3** shows the XRD patterns of the MX-T membrane. The MX-T membrane displays a broad diffraction peak centered at approximately 2θ=18.2^°^, which is attributed to the amorphous structure of the polyethersulfone (PES) support. The characteristic (002) diffraction peak at 8.1^°^ observed in the MX-T membrane indicates successful deposition of the Ti3C2Tx MXene layer on its surface. The persistence of the broad PES peak, together with the relatively weak MXene reflection, is expected for a thin surface coating, as the ultrathin MXene layer contributes only a small fraction of the total diffracted intensity, while the much thicker polymer support dominates the diffraction signal.

**Figure S2.** XRD patterns of the Ti_3_AlC_2_ MAX phase and the synthesized Ti_3_C_2_T_x_ MXene.

**Figure S3.** XRD patterns of the MX-T membrane.

**S3. TEM of Ti_3_C_2_T_x_ MXene**

TEM images collected at different magnifications show the morphology and crystalline structure of the prepared Ti_3_C_2_T_x_ MXene. Well-defined lattice fringes are consistently observed across different regions of the sample, demonstrating its crystalline structure.

**Figure S4**. TEM images of the prepared Ti3C2TX MXene at different magnifications, showing the morphology and well-defined lattice fringes of the MXene structure.

**S4. Transport Parameters of Synthesized Membranes**

The AL-DS configuration was employed to determine the water permeability coefficient (A) and the salt permeability coefficient (B). Sodium chloride solutions of varying concentrations (0.5-2.0 M, with 0.5 M increments) were used as the draw solution, and deionized (DI) water was used as the feed solution. In the AL-DS configuration, ICP arises exclusively from the reverse salt flux into the feed [2]. The water flux through the membranes can be expressed as:

$$J_{w}=A{\Delta\pi}_{e}$$

(S1)

where A is the water permeability coefficient, and Δπ_e_ is the effective osmotic pressure gradient across the selective layer. Dilutive external concentration polarization (ECP) happens when the permeate flow drags some draw solutes away from its surface. This reduces the solute concentration near the active layer and weakens the driving force for water transport [3,4]. To accurately evaluate the overall osmotic driving force across the selective layer, it is necessary to determine the solute concentration at the membrane surface on the draw side (C_D,m_). First, the Sherwood number is calculated using the following correlations for a rectangular channel:

$$Sh=1.85{(ReSc\frac{d_{h}}{L_{c}})}^{0.33} (Laminar flow)$$

(S2)

$$Sh=0.04{Re}^{0.75}{Sc}^{0.33} (Turbulent flow)$$

(S3)

$$Re=\frac{\rho d_{h}\nu}{\mu}$$

(S4)

$$Sc=\frac{\mu}{\rho D_{s}}$$

(S5)

where Re, Sc, d_h_, and L_c_ represent the Reynolds number, Schmidt number, hydraulic diameter, and channel length, respectively. In addition, D*_s_* denotes the solute diffusivity coefficient, μ is the dynamic viscosity, $\upsilon$the fluid velocity, and ρ the water density [4]. The values of μ, ρ, and D*ₛ* used in this study at 22 °C were obtained from our previous study. The solute mass transfer coefficient on the draw side (k) is related to the Sherwood number (Sh) according to:

$$Sh=\frac{kd_{h}}{D_{s}}$$

(S6)

Subsequently, the dilutive ECP modulus corresponding to each permeate flux ($J_{W}$) was calculated using the following expression:

$$\frac{C_{D,m}}{C_{D,b}}=\exp(-\frac{J_{W}}{K})$$

(S7)

Here, $C_{D,b}$ denotes the bulk draw solute concentration, while $C_{D,m}$represents the effective draw solute concentration at the membrane surface as influenced by dilutive ECP. J*_W_* corresponds to the permeate flux at each measurement step [5]. After determining the solute concentration at the membrane surface, the effective osmotic pressure, π*_D,e_* (bar), was estimated using the van’t Hoff relation:

$$\pi_{D,e}=nC_{D,m}RT$$

(S8)

In this equation, $n$ represents the solute dissociation number, $T$ (K) is the absolute temperature, and $R$ (8.314 J·mol⁻¹·K⁻¹) denotes the universal gas constant [6]. The water permeability coefficient (*A*) of the membranes was obtained by plotting the water flux against the effective osmotic pressure gradient, with the slope determined according to Eq. (1) [5]. The membrane selectivity was subsequently calculated using:

| $J_{s}=B(C_{D,m}-C_{F,m}$) | (S9) |
| --- | --- |

This equation relates the experimentally measured reverse salt flux ($J_{s}$) to the effective salt concentration gradient across the membrane. In this expression, B denotes the solute permeability coefficient, while C*_D,m_* and C*_F,m_* correspond to the effective solute concentrations at the selective layer interface on the draw and feed sides, respectively [7]. The solute permeability coefficient was determined by normalizing the reverse salt flux with respect to the effective draw solution concentration using [5]:

$$B=\frac{J_{s}}{C_{D,b}exp(-\frac{J_{w}}{k})}$$

(S10)

Here, k denotes the solute mass-transfer coefficient, calculated for a rectangular flow cell. The bulk solute concentrations of the feed and draw solutions were determined from conductivity measurements obtained using a conductometer (AR50 Accumet Research, Fisher Scientific)

**S5. Analytical Methods**

The quantitative analysis of PFOA and its short-chain products was performed using an Agilent 1290 Infinity II UHPLC system coupled to an Agilent 6495 triple quadrupole mass spectrometer (QQQMS; Agilent Technologies Inc., Canada). An Agilent Poroshell 120 EC-C18 (2.1 × 50 mm, 1.9 μm) column was used for chromatographic separation with mobile phases of water with 0.1% acetic acid (A) and acetonitrile with 0.1% acetic acid (B). The elution gradient was 0- 1 min, 5% B; 1÷7 min, increased from 5% B to 95% B; 7.1min, decreased to 5% B and held at 5% B for 2 min to equilibrate the column, with a flow rate of 0.4 mL/min. The column temperature was maintained at 40 °C throughout the run. The samples were analyzed with an injection volume of 2 µL.

The QQQMS analysis was performed in negative electrospray ionization (ESI-) mode with the following source operating conditions: the dry and sheath gases were nitrogen at a flow rate of 11 L/min. The dry and sheath gas temperatures were set to 130 °C and 400 °C, respectively. The fragmentor voltage (FV) was 166 V, and the nebulizer pressure was set at 40 psi. Capillary and nozzle voltages were both set at 2000 V. The cell accelerator voltage was fixed at 4 V for all compounds. Data acquisition was controlled with MassHunter software (Agilent Technologies Inc., Canada) in dynamic multiple reaction monitoring (dMRM) mode, and data analysis was performed using Quantitative Analysis software (Agilent Technologies Inc., Canada). **Table S1** lists the multiple reaction monitoring (MRM) transitions for PFOA and its short-chain products. Instrumental detection limits (IDLs) and instrumental quantification limits (IQLs) for PFOA and its short-chain products were established using direct injection of a standard mixture into pure methanol. The IDLs and IQLs shown in **Table S2** correspond to the lowest concentrations that achieve signal-to-noise (S/N) ratios of 3 and 10, respectively.

**Table S1.** MRM transitions of PFOA and its short-chain products in LC-QQQMS analysis.

| Compound Name | Precursor ion (m/z) | Production (m/z) | Collision Energy (V) | Polarity |
| --- | --- | --- | --- | --- |
| C_7_F_15_COOH | 413 | 369 | 8 | Negative |
|  | 413 | 169 | 16 | Negative |
| C_6_F_13_COOH | 363 | 319 | 8 | Negative |
|  | 363 | 169 | 16 | Negative |
| C_5_F_11_COOH | 313 | 269 | 8 | Negative |
|  | 313 | 119 | 16 | Negative |
| C_4_F_9_COOH | 263 | 219 | 8 | Negative |
| C_3_F_7_COOH | 213 | 169 | 8 | Negative |
| C_2_F_5_COOH | 163 | 119 | 8 | Negative |
| CF_3_COOH | 113 | 69 | 8 | Negative |

**Table S2.** Instrumental detection limits (IDLs) and instrumental quantification limits (IQLs) of PFOA and its short-chain products in LC-QQQMS analysis.

| Compound  Name | IDLs  (μg/L) | IQLs  (μg/L) |
| --- | --- | --- |
| C_7_F_15_COOH | 0.017 | 0.057 |
| C_6_F_13_COOH | 0.011 | 0.045 |
| C_5_F_11_COOH | 0.015 | 0.056 |
| C_4_F_9_COOH | 0.014 | 0.046 |
| C_3_F_7_COOH | 0.018 | 0.069 |
| C_2_F_5_COOH | 0.022 | 0.077 |
| CF_3_COOH | 0.294 | 0.992 |

Mineralization of the PFOA was assessed by measuring the carbon content of the samples at various intervals using a TOC analyzer (Shimadzu Corporation, Analytical & Measuring Instrument Division, Jiangsu, China). The percentage of TOC removal was subsequently calculated using:

$TOC removal \left( \% \right)=\frac{\left( {TOC}_{0}-TOC_{t} \right)}{{TOC}_{0}} \times100$

(S11)

where ${TOC}_{0}$ and $TOC_{t}$represent the initial TOC and the TOC at time t, respectively. The results from the PFOA degradation and TOC reduction were analyzed using the pseudo-first-order kinetic model, and the rate constants were evaluated based on the following reactions:

$$-\ln\left( \frac{TOC}{{TOC}_{0}} \right)=K_{app\left( TOC \right)}t$$

(S12)

where $K_{app\left( TOC \right)}$is the apparent reaction rate constant determined through curve fitting of the TOC decay analysis. The concentration of fluoride ions (F⁻) was measured using the DIONEX ICS-5000 Ion Chromatography system paired with the DIONEX ASRS conductivity detector. A 50-μL sample was injected by the integrated DIONEX AS-AP autosampler, with the fluoride (F⁻) ions detected and quantified using a DIONEX AS4A-SC anion-exchange column (25 cm × 4 mm (i.d)) eluted with 25 mM KOH at a flow rate of 1 mL/min.

The specific energy consumption, measured in kilowatt-hours (kWh) per gram of TOC decay (g TOC), is defined as the amount of electrical energy utilized and is calculated using:

$$SEC\left( kWh\left( {g TOC)}^{-1} \right) \right)=\frac{E_{cell}I t}{V_{t} \Delta\left( TOC \right)_{t}}$$

(S13)

where $t$ (h) represents time, and $I$ (A) is current.

To evaluate the physical stability of the coated layer, a dynamic leaching test was conducted by immersing the modified membranes in deionized water under continuous agitation. At predetermined time intervals, aliquots of the solution were collected and analyzed for titanium (Ti) concentration using an inductively coupled plasma–optical emission spectrometer (ICP-OES) (Thermo Scientific iCAP Pro Duo, Thermo Fisher Scientific, Cambridge, UK).

**S5. Mechanism of PFOA Mineralization**

Degradation in the MXene-based system proceeds via a dual-pathway anodic oxidation mechanism at the interface of the electroconductive membrane. Because forward osmosis concentrates PFOA at the membrane surface, direct anodic oxidation is the primary driver. The PFOA molecule transfers an electron directly to the electroconductive MXene anode, initiating the breakdown by forming a reactive radical. Working in tandem with this is a potent indirect oxidation pathway. The applied current naturally causes water to discharge at the anode, generating a high localized concentration of hydroxyl radicals •OH. While bulk PFOA resists •OH attack, these radicals rapidly react with the unstable intermediates formed during the initial direct oxidation step. Together, the direct electron transfer and the •OH attack systematically strip away CF_2_ units one by one until the molecule is mineralized.

The overall degradation is represented as follows:

PFOA (C_7_F_15_COOH) → C_6_F_13_COOH + 2F^‒^ → C_5_F_11_COOH + 2F^‒^ → … → CO_2_ + H_2_O + nF^‒^	(S14)

i. **Initiation (Anodic Electron Transfer):** The cycle begins when a PFOA molecule surrenders a single electron directly to the anode, generating an unstable perfluorocarboxylate radical:

C_7_F_15_COOH → C_7_F_15_COO^•^ + e⁻	(S15)

ii. **Decarboxylation:** This newly formed radical rapidly sheds a carbon dioxide molecule, leaving behind a perfluoroalkyl radical:

C_7_F_15_COO^•^ → C_7_F_15_^•^ + CO_2_	(S16)

iii. **Hydroxyl Radical Attack:** The abundant ^•^OH radicals generated by anodic water discharge react instantly with the perfluoroalkyl radical to form a perfluoroalcohol intermediate:

C_7_F_15_^•^ + ^•^OH → C_7_F_15_OH	(S17)

iv. **Intramolecular rearrangement and hydrolysis:** The highly unstable perfluoroalcohol immediately breaks down via rearrangement and hydrolysis. This step cleaves a CF_2_ unit, releasing fluoride ions and yielding a perfluorocarboxylate intermediate shortened by one carbon atom:

C_7_F_15_OH → C_6_F_13_COO− + by-products (e.g., F^‒^)	(S18)

v. **Chain Degradation (The Repeating Cycle):** The resulting shorter-chain intermediate C_6_F_13_COO^−^ re-enters the exact same loop-undergoing decarboxylation, •OH attack, and hydrolysis—to yield progressively shorter fragments:

C_6_F_13_COO^−^ → C_6_F_13_^•^ + CO_2_	(S19)

C_6_F_13_^•^ + ^•^OH → C_6_F_13_OH	(S20)

C_6_F_13_OH → C_5_F_11_COO^−^ + by-products (e.g., F^‒^)	(S21)

vi. **Final mineralization:** This cascade repeats continuously, knocking off carbon and fluorine atoms step-by-step until the carbon backbone is completely mineralized into safe, basic end products:

Intermediate → CO_2_ + H_2_O + F^‒^	(S22)

**S5. Removal of short-chain carboxylic PFAS**

**Figure S4** illustrates the water flux and Perfluoropentanoic acid (PFPeA) rejection for TFC and MX-T membranes in AL-FS mode. For both membranes, water flux gradually declines over time, indicating draw-solution dilution. PFOA rejection remains consistently high (~99-100%) for both membranes throughout the experiment, demonstrating stable separation performance, with TFC achieving 99.23-99.41% and MX-T achieving 99.43-99.50%. This indicates that although permeability changes over time, PFOA selectivity remains high, highlighting the robustness of both membrane systems in removing shorter-chain PFAS.

**Figure S5**. The water flux and short-chain PFOA rejection for TFC and MX-T membranes in AL-FS mode*.*
